# Pretrained Financial Language Model-Guided Multimodal Sensing with Hardware Provenance and Cross-Frequency Temporal Alignment for Event Prediction

**DOI:** 10.3390/s26175330

**Published:** 2026-08-22

**Authors:** Siyu Chen, Zhenrui Tian, Chenyan Zhu, Ruoyao Liu, Xianglong Pan, Jiahang Han, Yan Zhan

**Affiliations:** 1 China Agricultural University, Beijing 100083, China; 2 Peking University, Beijing 100871, China; 3 Tsinghua University, Beijing 100084, China

**Keywords:** artificial intelligence-driven sensing, multimodal sensing, pretrained financial language model, cross-modal consistency, hardware reliability perception

## Abstract

Financial media risk is jointly driven by multisource content, including news reports, corporate announcements, social media posts, short videos, and livestreams, while content authenticity, propagation velocity, asset relevance, and trading infrastructure conditions can simultaneously influence short-term market fluctuations. To address the limitations of existing methods, including their reliance on either textual information or market sequences, insufficient source verification, and inadequate alignment of asynchronous multimodal signals, FMRP-Net is proposed for artificial intelligence-driven sensing. Event semantics, risk categories, and asset association information are first extracted through a pretrained financial language model and cross-modal consistency analysis. A dual-layer hardware reliability perception module is then employed to integrate sensing evidence from cameras, microphones, terminal inertial signals, server temperature, power consumption, network traffic, and transmission latency. Heterogeneous temporal propagation graphs, cross-frequency alignment, and bidirectional propagation–market coupling are further incorporated to jointly predict market direction, volatility, risk level, and propagation trends. Experimental results demonstrate that FMRP-Net achieved an Accuracy of 0.832, a Macro-F1 of 0.824, a ROC-AUC of 0.891, an MCC of 0.665, and a PR-AUC of 0.883 for market direction prediction over future horizons of 5, 15, 30, and 60 min, indicating a balanced performance in terms of Precision and Recall. For volatility prediction, MAE, RMSE, and MAPE values of 0.0178, 0.0271, and 12.46% were obtained, respectively, together with an $R^{2}$ of 0.812. In the ablation study, the media risk Macro-F1 and source reliability AUC reached 0.842 and 0.929, respectively, while the propagation-scale prediction error was reduced to 0.109 and the average early-warning lead time reached 10.6 min. These results demonstrate that the integration of multimedia semantics, hardware sensing evidence, and propagation structures can effectively improve the accuracy, stability, and interpretability of financial market prediction and risk early warning.

## 1. Introduction

With the rapid development of digital data, mobile Internet technologies, and intelligent trading systems, the mechanisms governing information generation, dissemination, and market feedback have undergone fundamental changes [1]. Algorithmic and high-frequency trading accelerate the transmission of risk information to prices, trading volumes, and market volatility [2], while information has expanded far beyond historical prices and conventional news to include social media, short videos, livestreams, images, speech, subtitles, and user comments [3]. Following major policy announcements, regulatory actions, or unexpected public events, multimodal information can spread rapidly through recommendation algorithms and user interactions, influencing investor sentiment, market attention, and herding behavior, ultimately triggering abnormal trading activities and increased volatility [4]. Consequently, accurately perceiving the content characteristics, propagation dynamics, and market impacts of risk information has become essential for risk management and intelligent investment decision-making [5]. The market impact of media depends not only on textual sentiment but also on information authenticity, source credibility, asset relevance, cross-modal consistency, and propagation dynamics [6]. Manipulated headlines, reused historical materials, audio-visual inconsistencies, and misleading subtitles may substantially alter investor perception [7], while hardware-level indicators such as network traffic, server load, power consumption, and resource utilization can reflect the trading pressure induced by risk events [8]. Risk prediction should, therefore, be formulated as a multimodal sensing problem that jointly models multimedia content, information diffusion, hardware operating states, and market dynamics [9]. From a sensing perspective, financial risk prediction can be viewed as an inference process built on heterogeneous observations collected from both content sources and supporting infrastructures. Physical acquisition traces can provide evidence about content provenance and authenticity, while infrastructure telemetry, such as network traffic, server load, power consumption, and resource utilization, captures the system-level response associated with information dissemination and trading activity. When combined with semantic, propagation, and market observations, these heterogeneous sensing signals provide complementary evidence for assessing the reliability and potential impact of risk-related events. Their joint modeling, therefore, extends financial risk analysis beyond conventional price- or sentiment-based forecasting toward an AI-driven sensing framework in which heterogeneous measurements are adaptively interpreted and integrated for downstream risk assessment. Existing forecasting methods mainly consist of statistical and machine-learning models [10]. Traditional statistical approaches rely on assumptions such as linearity and stationarity [11], limiting their ability to model highly nonlinear and dynamic markets [12]. Conventional machine-learning methods improve nonlinear modeling but still depend heavily on handcrafted features and have limited capability to capture complex semantics and long-range dependencies [13]. Although recent multimodal sentiment analysis and information propagation studies have achieved promising results, most assume that media content is authentic and rarely consider cross-modal inconsistencies such as image–text mismatches, manipulated audio, or reused historical materials. Likewise, propagation models primarily focus on diffusion patterns while insufficiently characterizing their influence on specific assets. Furthermore, multimedia events, propagation records, hardware sensing signals, and high-frequency market sequences exhibit substantially different temporal granularities, making direct fusion prone to temporal misalignment, feature redundancy, and accumulated noise, thereby limiting prediction accuracy and robustness.

In recent years, deep learning has provided new technical pathways for market prediction [14]. Recurrent neural networks and long short-term memory networks can capture long-term dependencies in price sequences, temporal convolutional networks can extract local variation patterns at different scales through dilated convolutions, and Transformer-based models can use self-attention mechanisms to model long-range dependencies while improving parallel computation and multivariate sequence modeling [15]. In multimodal analysis, pretrained language models can extract events, sentiment, and asset associations from text; visual, audio, and video encoders can identify image content, speech characteristics, and temporal video changes; and graph neural networks and temporal graph networks can represent propagation relationships among users, content, devices, platforms, and assets [16]. Nevertheless, existing deep-learning studies still largely rely on single-modality modeling or simple feature concatenation and lack a unified representation of multimedia authenticity, hardware-source reliability, propagation dynamics, and market responses [17]. Fixed fusion weights also fail to adapt effectively to changing market conditions. When one modality is missing, distorted, or contaminated by noise, prediction performance and stability may deteriorate substantially [18]. Odunaike et al. [19] developed a real-time data-driven framework for risk modeling and portfolio decision-making by integrating market quotations, macroeconomic indicators, news sentiment, social media information, and transaction data with online learning, temporal convolutional networks, and ensemble forecasting. The Sharpe ratio was increased from 0.92 to 1.35, the maximum drawdown was reduced by 50%, and the market-shock response speed was improved by more than 95%. Yang et al. [20] proposed the TFT-ASRO model, which integrates price, trading-volume, and market-sentiment data to predict returns, volatility, and the Sharpe ratio through multi-task learning, adaptive optimization, and uncertainty quantification. An MAE of 0.152, an RMSE of 0.197, a correlation coefficient of 0.723, and a directional accuracy of 73.4% were achieved. Akhavanpour et al. [21] developed an adaptive stock-prediction framework based on Kafka and microservices, in which convolutional neural network (CNN), gated recurrent unit (GRU), and long short-term memory network (LSTM) models were compared in real time and the best-performing model was dynamically selected. Maximum $R^{2}$ values of 0.97, 0.96, and 0.94 were obtained for EUR/USD, Google, and Microsoft, respectively, thereby improving predictive adaptability and system scalability. Zhu et al. [22] proposed a stock-price prediction framework integrating principal component analysis, anomaly detection, correlation analysis, and LSTM. By compressing multidimensional indicators and learning long-term temporal dependencies, a cumulative explained variance exceeding 95% was obtained for the leading principal components, while the correlation coefficient between CP and GPM reached 0.96, providing support for market risk analysis and investment decision-making.

To address these limitations, a short-term market prediction framework termed FMRP-Net is proposed by integrating multimedia risk propagation, hardware sensing evidence, and market sequences. Large language models together with image, audio, and video encoders are first employed to extract event topics, target assets, sentiment polarity, risk types, impact intensity, and semantic uncertainty. Consistency relationships between headlines and bodies, text and images, video and speech, and speech and subtitles are subsequently analyzed. Camera pattern noise, microphone spectral fingerprints, terminal motion states, and compression traces are then used to evaluate content-source reliability. Meanwhile, server power consumption, temperature, resource utilization, network latency, and access traffic from media platforms and trading infrastructures are incorporated to sense platform propagation pressure and trading operating states induced by risk events. Finally, a heterogeneous dynamic graph comprising content, users, devices, platforms, and assets is constructed. Cross-frequency temporal alignment, temporal graph neural networks, bidirectional cross-attention, and dynamic gated fusion are employed to jointly predict risk-information propagation trends, market directions, future volatility, and market risk levels. The main contributions are summarized as follows.

1. Short-term market prediction is reformulated from a multimodal intelligent sensing perspective, in which text, images, audio, video, propagation behavior, hardware sensing data, and market sequences are incorporated into a unified analytical framework, extending beyond conventional paradigms that rely primarily on prices and textual sentiment.
2. A pretrained-financial-language-model-driven multimedia risk consistency perception module is proposed to identify exaggerated headlines, image–text mismatches, audio–visual conflicts, subtitle contradictions, and content uncertainty while extracting event semantics and sentiment information. The novelty lies in coupling domain semantics to physical provenance, cross-modal contradiction evidence, and asset-specific downstream gating, rather than in claiming a new language-model backbone.
3. A dual-layer hardware reliability perception mechanism is designed. Signals from content-capture devices are used to assess source authenticity, while sensing signals from media platforms and trading infrastructures are used to characterize propagation pressure, trading-request density, and system congestion.
4. A media propagation–market response coupling prediction module is constructed. A heterogeneous dynamic graph and a cross-frequency temporal alignment mechanism are used to address asynchronous associations among event-driven media signals, minute-level propagation data, second-level hardware signals, and high-frequency market sequences.
5. Dynamic reliability weighting and multi-task learning mechanisms are introduced to jointly perform content-risk recognition, source-reliability assessment, propagation-trend prediction, and short-term market forecasting, thereby improving prediction stability and risk early-warning capability under abrupt events, modality missingness, sensing noise, and changing market conditions.

## 2. Related Work

### 2.1. Media Analysis and Multimedia Risk Content Identification

The objective of media analysis is to transform unstructured information, including news, corporate announcements, social media, and investor comments, into structured semantic representations for identifying events, sentiment, entities, and potential market impacts [23]. Research has evolved from lexicon-based and traditional machine-learning methods toward deep neural networks and domain pretrained language models [24], enabling more accurate understanding of domain-specific terminology and implicit semantic relationships [25]. More recently, large language models have further extended media analysis from sentiment classification to event extraction, entity linking, risk attribution, and market impact analysis, providing richer semantic representations for downstream applications [26,27]. Meanwhile, risk information has expanded beyond textual reports to multimedia content consisting of images, audio, video, subtitles, and user comments [28]. Existing multimodal analysis methods detect inconsistencies across modalities by modeling semantic alignment between text, images, speech, and video, allowing the identification of manipulated or misleading multimedia content such as image–text mismatches, audio–visual inconsistencies, and edited videos [29,30]. However, these methods are primarily designed for misinformation detection or content moderation [31], while media analysis generally assumes that multimedia information is authentic and rarely considers how cross-modal inconsistencies influence asset returns, volatility, or risk [32]. Therefore, the proposed framework jointly models semantics, cross-modal consistency, information uncertainty, and asset relevance, enabling multimedia information to contribute directly to market prediction and risk assessment.

### 2.2. Hardware-Based Source Attribution and Infrastructure State Sensing

Hardware-based source attribution exploits physical traces embedded in multimedia content to determine acquisition devices, content provenance, and post-processing operations [33]. Representative techniques include camera sensor fingerprints, microphone characteristics, motion-sensor signals, and video encoding artifacts, which enable the detection of device inconsistency, re-recording, screen recapture, and multimedia manipulation [34,35]. Meanwhile, infrastructure state sensing monitors the operating status of computing and communication systems through network conditions, platform traffic, and server telemetry, providing indicators of information dissemination pressure, computational load, and trading-system congestion [36,37]. Although these hardware signals are widely applied in multimedia forensics and infrastructure monitoring, existing studies generally treat them as independent problems and rarely exploit them as sensing information for market prediction [38,39]. Therefore, the proposed framework jointly models content-source reliability and infrastructure states, enabling hardware information to characterize media credibility, market attention, information propagation pressure, and trading congestion for risk analysis.

### 2.3. Risk Information Propagation and Market Prediction

Risk information propagation modeling aims to characterize how information diffuses across users and platforms over time [40]. Existing methods include propagation trees for describing diffusion structures, temporal point processes for modeling propagation dynamics, and graph neural networks for learning evolving relationships among users, content, and platforms [41,42]. These approaches effectively predict information diffusion patterns, such as propagation scale and speed, but rarely investigate their influence on the market responses of specific assets [43]. Market prediction has evolved from sequential models such as LSTM and temporal convolutional network (TCN) to Transformer-based architectures and multimodal learning frameworks that integrate market data with news and social media information [44]. Although these models improve the prediction of market trends and volatility, they still rely primarily on price sequences and textual sentiment, with limited consideration of information authenticity, propagation structures, hardware provenance, and infrastructure states. Consequently, existing studies fail to establish a complete mechanism linking risk-information diffusion to subsequent trading behavior and market volatility [45]. Therefore, the proposed framework constructs a heterogeneous dynamic graph connecting content, users, devices, platforms, and assets, and employs cross-frequency temporal alignment and dynamic fusion to jointly model risk-information propagation and short-term market responses.

## 3. Materials and Method

### 3.1. Data Collection

Data were collected from January 2023 to December 2025, covering multimedia content, information propagation behavior, hardware signals associated with content sources, operating states of media and trading infrastructures, and market sequences, as shown in Table 1. Textual data were obtained from stock exchange disclosure platforms, publicly accessible regulatory websites, official websites of listed companies, major news platforms, and public social media interfaces. The collected content included news headlines, article bodies, corporate announcements, regulatory documents, social media posts, and investor comments. Metadata such as publication time, publishing platform, source account, interaction status, involved companies, and associated assets were retained simultaneously. Image, audio, and video data were primarily obtained from public news webpages, official corporate communication channels, short-video platforms, and livestreaming channels. Controlled experiments were also conducted to supplement samples involving original acquisition, repeated compression, screen recapture, audio re-dubbing, and cross-device recording. During image and video acquisition, information including resolution, exposure settings, frame rate, encoding format, quantization parameters, and compression frequency was retained. For audio data, the sampling rate, gain, spectral distribution, and background noise were recorded. Terminal inertial signals were synchronously collected during multimedia acquisition to analyze the temporal consistency among device posture, vibration states, and motion patterns in video frames. Propagation behavior was continuously collected through public platform interfaces and scheduled acquisition programs. Reposting, commenting, liking, propagation depth, propagation breadth, user relationships, and cross-platform migration were updated at one-minute intervals. Dynamic propagation graphs were subsequently constructed according to publishing, reposting, commenting, device-generation, and asset-association relationships. Infrastructure operating data were obtained from local media acquisition nodes, market-data access servers, network switching devices, and experimental trading gateways. The collected variables included processor and server temperatures, power consumption, current, voltage, memory utilization, disk input/output, network traffic, request volume, transmission latency, jitter, and packet loss. Different operating signals were continuously recorded at second-level or higher frequencies according to their variation rates to characterize platform access pressure, market-data request density, and system congestion following abrupt events. Market data were obtained from authorized stock exchange quotation interfaces and professional databases, covering individual equities, industry indices, and major market indices. The recorded variables included prices, trading volume, turnover, bid–ask spreads, order-book depth, and relevant technical indicators. All data were matched using unified timestamps. Observation and prediction intervals were established around the publication time of each event, resulting in event-level samples organized in the form of “media event–associated asset–observation window.” These samples were used for content risk identification, source reliability assessment, propagation trend prediction, and short-term market forecasting.

The source and sample-construction protocol was specified as follows. Corporate disclosures were retrieved from the official Shanghai Stock Exchange and Shenzhen Stock Exchange announcement portals and CNINFO; regulatory releases were retrieved from the China Securities Regulatory Commission website; licensed quotations and corporate-action adjustments were obtained through the Wind financial-data interface and the two exchange quotation interfaces. Public news and multimedia records were collected from the public webpages or documented developer interfaces of Xinhua Finance, Sina Finance, Eastmoney, Weibo, and Bilibili. For every record, the publisher timestamp, platform identifier, retrieval timestamp, canonical URL hash, source-account identifier, and associated security code were retained. Times were converted to China Standard Time (UTC+8), and collection nodes were synchronized by NTP; the earliest publisher timestamp verified from the canonical record was used as the event time $t_{0}$. Duplicate URL hashes, verbatim or near-duplicate text with cosine similarity above 0.95 within 24 h, records lacking a verifiable timestamp, and records without an asset association score of at least 0.60 were excluded. Derivative reposts were retained as propagation edges but were not treated as independent root events. Each sample used only observations in $\left[t_{0}-120\;\min,t_{0}\right]$, with targets constructed at 5, 15, 30, and 60 min after $t_{0}$. Events sharing the same root URL, disclosure identifier, or near-duplicate cluster were assigned to the same chronological split.

Hardware telemetry was measured in the authors’ controlled acquisition and market-access environment rather than inferred from nominal rates. HPE iLO/IPMI counters were polled every second for server temperature and utilization; the APC AP8959 smart PDU was polled every two seconds for power, current, and voltage; Intel X710 interface counters and active probes supplied one-second traffic, latency, jitter, and packet-loss measurements. Device clocks used the same NTP source as the content collectors. Samples with failed checksums, impossible sensor ranges, duplicate timestamps, or clock offsets exceeding 200 ms were removed; gaps were recorded explicitly and were not forward-filled before reliability estimation. Accordingly, Table 1 reports actual retained records after cleaning, not theoretical sampling-rate products. The apparently round figures in the original version were nominal maxima and have been replaced by measured retained counts.

### 3.2. Data Augmentation

The fundamental principle of data augmentation is to apply random transformations within reasonable ranges to the original data without altering the core semantics, event attributes, or prediction labels of the samples, thereby generating extended samples that approximate realistic application scenarios. Because the multimedia content, hardware sensing sequences, propagation relationships, and market data considered in this study are affected by different noise sources and missing-data patterns, identical augmentation strategies cannot be applied across all modalities. Let the original data of the *m* -th modality be denoted by $x_{m}$, the augmentation operator by $T_{m}\left(\cdot \right)$, and the corresponding random parameter by $\theta_{m}$. The augmented sample can then be expressed as

(1) $${\tilde{x}}_{m}=T_{m}\left(x_{m};\theta_{m}\right),\;y\left({\tilde{x}}_{m}\right)=y\left(x_{m}\right),$$

where ${\tilde{x}}_{m}$ denotes the augmented modal data, and *y* denotes the media authenticity, associated asset, propagation state, or market prediction label corresponding to the sample. This constraint ensures that augmentation operations simulate only realistic variations in expression, acquisition noise, and observation missingness, while preserving the factual attributes of event and its relationship with the target asset. For textual data, synonym-based paraphrasing, random masking, and headline perturbation are applied to news, social media posts, video subtitles, and user comments. Random masking hides noncritical words with a predefined probability, thereby reducing excessive dependence on isolated keywords. Let the textual sequence be denoted by $X=w_{1},w_{2},\ldots ,w_{n}$. The random masking process is formulated as

(2) $${\tilde{w}}_{i}=\left\{\begin{array}{cc} {}[\mathrm{MASK}], & m_{i}=0,\\ w_{i}, & m_{i}=1, \end{array}\right.\;m_{i}∼\mathrm{Bernoulli}\left(1-p_{t}\right).$$

where $p_{t}$ denotes the word-masking probability. Critical entities, including company names, stock codes, event timestamps, monetary amounts, and policy names, are excluded from random replacement. Headline perturbation is restricted to changes in word order, syntactic structure, and sentiment intensity. The entity set *E* and event-relation set *R* are required to remain unchanged before and after augmentation, satisfying $E\left(\tilde{X}\right)=E\left(X\right)$ and $R\left(\tilde{X}\right)=R\left(X\right)$. This restriction prevents the generation of erroneous text that is inconsistent with the original event. For image and video data, augmentation operations are designed to simulate visual variations caused by heterogeneous terminals, platform compression, and dissemination environments. These operations include random cropping, brightness adjustment, local occlusion, video compression, and frame dropping. Let the image at the *t*-th frame be denoted by $I_{t}$. The augmentation process is expressed as

(3) $${\tilde{I}}_{t}=d_{t}Q_{c}\left[M_{t}⊙C\left(\gamma I_{t}+\beta \right)\right],\;d_{t}∼\mathrm{Bernoulli}\left(1-p_{f}\right),$$

where C(·) denotes the cropping operation, γ and β control brightness scaling and intensity shifting, respectively, $M_{t}$ denotes a local occlusion mask, ⊙ represents element-wise multiplication, $Q_{c}\left(\cdot \right)$ denotes video encoding with compression quality *c*, and $p_{f}$ denotes the frame-dropping probability. Regions required for identifying event subjects, scenes, and device provenance are preserved during augmentation. Consequently, company logos, principal individuals, and critical visual evidence associated with content authenticity are not removed through cropping or occlusion. For audio data, environmental noise addition, volume adjustment, slight speed perturbation, and compression are adopted to simulate variations introduced by livestream recording, mobile-device acquisition, and secondary platform encoding. Let the original audio signal be represented by a(t). The augmented audio signal is defined as

(4) $$\tilde{a}\left(t\right)=Q_{a}\left[\kappa a(\rho t)+\lambda n(t)\right],$$

where κ denotes the volume-scaling coefficient, ρ denotes the playback-speed perturbation factor, n(t) represents environmental noise, λ controls the noise intensity, and $Q_{a}\left(\cdot \right)$ denotes the audio compression operator. Speed and noise perturbations are constrained within reasonable ranges to ensure that speech content, speaker identity, and event semantics remain recognizable. Meanwhile, source-forensic characteristics, such as microphone spectral fingerprints, are retained to the greatest extent possible. For sensing data generated by servers, network nodes, and mobile terminals, random channel missingness, temporal cropping, drift simulation, sampling-interval perturbation, and low-amplitude noise injection are employed. Let the observation of the *k*-th sensing channel at time *t* be denoted by $s_{k}\left(t\right)$. The augmented signal is formulated as

(5) $${\tilde{s}}_{k}\left(t\right)=m_{k}\left(t\right)\left[s_{k}\left(t+\Delta t\right)+b_{k}t+\epsilon_{k}\left(t\right)\right],$$

where $m_{k}\left(t\right)∼\mathrm{Bernoulli}\left(1-p_{k}\right)$ denotes the channel-availability mask, Δt represents sampling-time perturbation, $b_{k}t$ denotes temporally accumulated sensor drift, and $\epsilon_{k}\left(t\right)∼N\left(0,\sigma_{k}^{2}\right)$ represents low-amplitude random noise. This strategy simulates missingness, drift, and asynchronous sampling commonly observed in power consumption, temperature, network latency, and resource-utilization measurements, thereby improving model adaptability to unstable hardware environments. For risk-information propagation graphs, incomplete observation scenarios are simulated through the removal of noncritical nodes, random masking of propagation edges, and truncation of early propagation processes. Let the original propagation graph be represented by G=(V,E). The augmented graph is defined as

(6) $${\tilde{G}}_{\tau }=\left(V\setminus V_{d},\;\left\{\left(u,v,t\right)\in E\setminus E_{d}|t\leq \tau \right\}\right),$$

where $V_{d}$ denotes the set of removed low-impact nodes, $E_{d}$ denotes the set of randomly masked propagation edges, and τ represents the cutoff time for early observation. By setting τ to 10%, 20%, and 30% of the complete propagation process, realistic early-warning conditions can be simulated in which only limited reposting and commenting records are available during the initial stage of an event. To preserve predictive consistency between original and augmented samples, a consistency constraint is incorporated in addition to the task-specific objective:

(7) $$L=L_{\mathrm{task}}+\lambda D_{\mathrm{KL}}\left(f\left(x\right),|,f\left(\tilde{x}\right)\right),$$

where $L_{\mathrm{task}}$ denotes the original prediction-task loss, f(·) represents the model output, $D_{\mathrm{KL}}$ measures the discrepancy between the predictive distributions of the original and augmented samples, and λ is the corresponding weighting coefficient. Through these augmentation strategies, robust representations can be learned against expression variations, platform compression, modality missingness, sensing noise, and incomplete propagation observations, while consistency in content authenticity, device provenance, associated assets, and market labels is maintained.

### 3.3. Proposed Method

#### 3.3.1. Overall

FMRP-Net is designed around the primary task of short-term market volatility prediction. Processed multimedia content, hardware sensing sequences, propagation relationships, and market time-series data are incorporated into a unified end-to-end framework, in which risk semantics extraction, information reliability assessment, propagation state modeling, and market response prediction are performed sequentially. First, textual content, images, audio signals, video keyframes, and subtitles are processed by a pretrained financial language model, a visual encoder, an audio encoder, and a video temporal encoder, respectively, to obtain modality-specific representations. Event topics, target companies, associated assets, sentiment orientations, risk categories, impact intensities, information certainty, and timeliness are extracted from textual content and automatic speech transcripts by the financial language model. Scene information, individuals, corporate logos, acoustic characteristics, and temporal visual changes are captured by the visual, audio, and video branches. Cross-modal attention is subsequently employed to compare the semantic relationships between headlines and article bodies, text and images, visual scenes and speech, speech and subtitles, and current materials and historical materials. These relationships are combined with event–asset matching scores to produce an integrated media risk representation containing event semantics, content conflicts, asset relevance, and modality uncertainty. The resulting representation is then transmitted to the dual-layer hardware reliability perception and temporal encoding module, where it is jointly analyzed with sensing sequences obtained from content-acquisition devices and infrastructure systems. At the content-source layer, camera pattern noise, video compression traces, microphone spectral fingerprints, and terminal motion states are encoded as device-source features. These features are used to assess whether images, audio signals, and videos originate from consistent devices and whether screen recapture, audio re-dubbing, or cross-device transcription has occurred, thereby producing a content-source reliability representation. At the infrastructure layer, continuous signals, including server temperature, power consumption, resource utilization, network traffic, latency, jitter, and packet loss, are processed through local temporal convolutions and long-range temporal encoders. Platform dissemination pressure and trading infrastructure operating pressure are consequently represented. Dynamic reliability weights are assigned according to the missingness, stability, anomaly magnitude, and historical consistency of each sensing channel, allowing channels affected by severe noise or abnormal operating conditions to contribute less during subsequent fusion.

The media risk representation, source reliability representation, and infrastructure pressure representation are subsequently transmitted to the media propagation–market response coupling prediction module. Processed content, users, devices, platforms, and assets are represented as heterogeneous node types, while publication, reposting, commenting, device generation, cross-platform migration, and asset association are represented as heterogeneous edge relations. Node states are aggregated along propagation paths through a Temporal Graph Network, yielding representations of the current propagation scale, growth velocity, propagation depth, and critical nodes. Meanwhile, historical prices, trading volumes, bid–ask spreads, order-book depths, and technical indicators are processed by a market temporal encoder to produce a multiscale market state representation. Because media events, propagation behavior, hardware signals, and market sequences exhibit substantially different temporal frequencies, cross-frequency alignment is performed through a unified prediction window, time-interval encoding, and event-decay mechanisms, ensuring that all branch representations correspond to the same market prediction time. During the fusion stage, media-to-market cross-attention is used to identify the influence of high-risk content, rapid propagation structures, and reliable sources on future market states. Reverse market-to-media attention is employed to characterize how price movements, trading activity, and market risk may stimulate subsequent discussion and information diffusion. A gating network further allocates branch-specific weights dynamically according to content credibility, asset relevance, propagation intensity, sensing reliability, and market conditions. When media content is credible and propagates rapidly, greater importance is assigned to the media propagation representation. When media information is sparse or its source is suspicious, increased reliance is placed on market sequences and infrastructure states. When platform traffic, trading-server loads, and market anomaly indicators change synchronously, the confidence assigned to abrupt-risk warnings is increased. Finally, the fused representation is delivered to multiple task-specific prediction heads. Market direction, realized volatility, and market risk level over different future horizons are produced by the primary-task heads, whereas media risk category, source reliability, future propagation scale, propagation velocity, and warning lead time are estimated by the auxiliary-task heads. Joint training is performed through shared intermediate representations, allowing content-risk identification and propagation-trend prediction to continuously constrain the market prediction branch. A complete methodological chain is thereby established from multimedia risk perception and hardware evidence verification to propagation process modeling and market response prediction.

#### 3.3.2. Pretrained-Financial-Language-Model-Driven Multimedia Risk Consistency Perception Module

**Figure 1:**
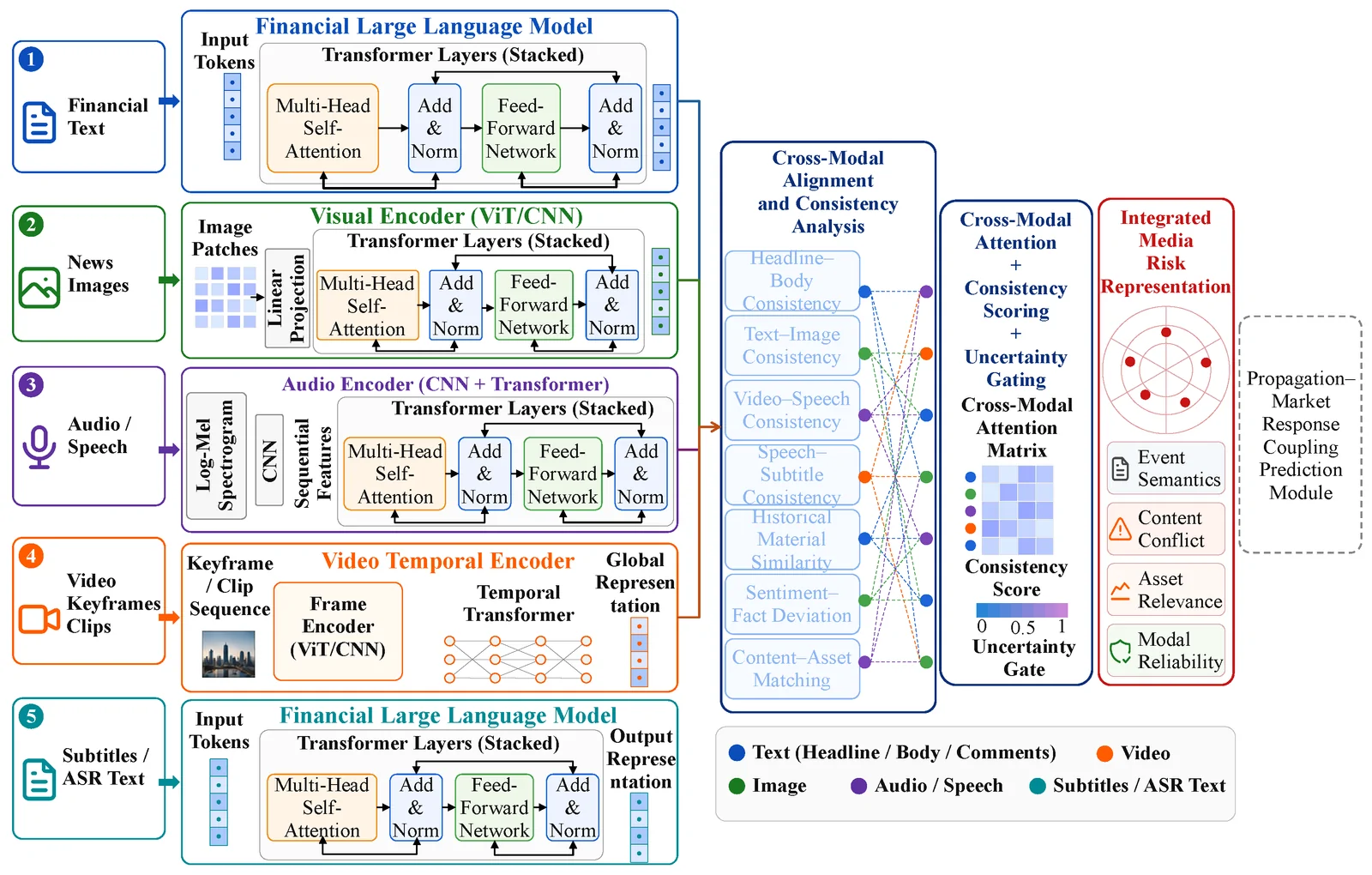
Architecture of the pretrained-financial-language-model-driven multimedia risk consistency perception module.

The pretrained-financial-language-model-driven multimedia risk consistency perception module receives processed text, images, audio, video, and subtitles as inputs and projects heterogeneous modalities into a unified risk semantic space. As shown in Figure 1, let the textual sequence be denoted by $X^{t}\in R^{N_{t}\times d_{t}}$, the image by $X^{v}\in R^{H_{v}\times W_{v}\times C_{v}}$, the audio spectrogram by $X^{a}\in R^{H_{a}\times W_{a}\times C_{a}}$, and the video by $X^{m}\in R^{T_{m}\times H_{m}\times W_{m}\times C_{m}}$. The textual branch is implemented using a BERT-scale financial domain model containing $L_{t}$ Transformer layers. Each layer consists of $h_{t}$ self-attention heads, a hidden representation of dimension $d_{t}$, and a feed-forward network with width $r_{t}d_{t}$. The representation at the *l*-th layer is defined as

(8) $$Z_{l}^{t}=\mathrm{LN}\left(Z_{l-1}^{t}+{\mathrm{MHA}}_{l}^{t}\left(Z_{l-1}^{t}\right)\right),\;{Z_{l}^{t}}^{\prime }=\mathrm{LN}\left(Z_{l}^{t}+{\mathrm{FFN}}_{l}^{t}\left(Z_{l}^{t}\right)\right).$$

Event topics, associated assets, sentiment polarity, impact intensity, uncertainty, and timeliness are extracted from the final textual state through task prompts and semantic query vectors. In the visual branch, each image is partitioned into patches of size $P_{h}\times P_{w}$, linearly projected, and processed by a visual Transformer containing $L_{v}$ layers with hidden width $d_{v}$. The audio branch employs a convolutional frontend with kernel size $K_{a}^{h}\times K_{a}^{w}$ and channel widths $C_{a}^{\left(1\right)},\ldots ,C_{a}^{\left(L_{a}\right)}$ to extract local time–frequency patterns, followed by an $L_{a}$-layer temporal Transformer for modeling long-range acoustic dependencies. In the video branch, spatial features are first extracted from individual frames, after which an $L_{m}$-layer temporal attention network is used to model shot transitions, human actions, and visual evolution. The outputs of different modalities are projected into a shared dimension $d_{c}$ through independent projection matrices:

(9) $${\hat{Z}}^{q}=Z^{q}W_{q}+b_{q},\;q\in t,v,a,m,s,$$

where *s* denotes the subtitle or speech-transcription modality. Specifically, the public ProsusAI/finbert checkpoint [46] and its unchanged tokenizer initialize the textual branch. It is not used off the shelf: all FinBERT layers are fine-tuned jointly with six task heads on the chronological training partition for event type, entity–asset link, sentiment, impact intensity, certainty, and timeliness. No validation or test text is included in this fine-tuning. The checkpoint identifier, preprocessing rules, random seeds, task-label schema, and training script are included in the reproducibility package; the fine-tuning corpus is described by source, date, event category, and label frequency, with a de-identified subset released where licensing permits. We, therefore, use “pretrained financial language model” throughout and do not rely on model scale as a novelty claim. To characterize cross-modal consistency, the textual event representation is treated as the query, whereas image, audio, video, and subtitle representations are treated as keys and values. The corresponding conditional representations are computed as

(10) $$A_{tq}=\mathrm{softmax}\left(\frac{{\hat{Z}}^{t}W_{Q}^{q}{\left({\hat{Z}}^{q}W_{K}^{q}\right)}^{T}}{\sqrt{d_{c}}}\right),\;R_{tq}=A_{tq}{\hat{Z}}^{q}W_{V}^{q}.$$

Consistency between headlines and article bodies, text and images, video and speech, and speech and subtitles is estimated jointly from matching vectors and difference vectors:

(11) $$c_{tq}=\sigma \left(w_{q}^{T}\left[{\bar{Z}}^{t};{\bar{R}}_{tq};\left|{\bar{Z}}^{t}-{\bar{R}}_{tq}\right|;{\bar{Z}}^{t}⊙{\bar{R}}_{tq}\right]+b_{q}\right).$$

Compared with cosine similarity alone, this structure can simultaneously characterize directional agreement, local discrepancies, and feature interactions. When two modalities are semantically consistent with respect to the same event, the difference term approaches zero and the interaction term is strengthened. When image–text objects or audio–visual events are mismatched, the difference term increases and $c_{tq}$ decreases. To identify reused historical images and previously published materials, the current visual or video representation is matched against a historical event memory bank M using temperature scaling:

(12) $$p_{\mathrm{reuse}}=\max_{u\in M}\frac{\exp\left(\mathrm{sim}\left({\bar{Z}}^{m},u\right)/\tau \right)}{\sum_{j\in M}\exp\left(\mathrm{sim}\left({\bar{Z}}^{m},j\right)/\tau \right)}.$$

The event representation is also matched bilinearly with candidate asset embeddings, preventing an asset from being associated incorrectly merely because a company name appears in the text:

(13) $$p\left(a_{j}|e\right)=\frac{\exp\left({\bar{Z}}^{tT}W_{e}E_{a_{j}}\right)}{\sum_{k}\exp\left({\bar{Z}}^{tT}W_{e}E_{a_{k}}\right)}.$$

The integrated media risk representation is generated from event semantics, cross-modal consistency, historical reuse probability, asset association probability, and modality uncertainty. Joint operation with the dual-layer hardware reliability perception and temporal encoding module is then performed. The source reliability vector $R_{h}$ produced by the hardware module does not replace semantic assessment; instead, it is used as a gating condition to regulate the contribution of each modality:

(14) $$g_{q}=\frac{\exp\left(\phi_{q}\left([{\bar{Z}}^{q};R_{h};c_{tq}]\right)\right)}{\sum_{j}\exp\left(\phi_{j}\left([{\bar{Z}}^{j};R_{h};c_{tj}]\right)\right)},\;F_{\mathrm{risk}}=\sum_{q}g_{q}{\bar{Z}}^{q}.$$

A clear stability property is provided by this design. Since $g_{q}\geq 0$ and $\sum_{q}g_{q}=1$, the fused representation remains within the convex hull formed by the modality-specific representations. Therefore,

(15) $${\left|F_{\mathrm{risk}}\right|}_{2}\leq \sum_{q}g_{q}{\left|{\bar{Z}}^{q}\right|}_{2}\leq \max_{q}{\left|{\bar{Z}}^{q}\right|}_{2}.$$

This inequality indicates that the fused representation cannot be amplified without bound by a single abnormal modality. When semantic conflicts, abnormal device provenance, or high uncertainty are detected in a modality, its gating weight is reduced, thereby limiting the influence of misleading sentiment and manipulated materials on market prediction. For the target task, this module determines what a event expresses, whether different modalities are mutually consistent, whether the event is associated with the target asset, and whether its physical source is reliable. A fact-constrained and asset-specific risk representation is consequently provided for the subsequent propagation–market response coupling prediction process.

#### 3.3.3. Dual-Layer Hardware Reliability Perception and Temporal Encoding Module

**Figure 2:**
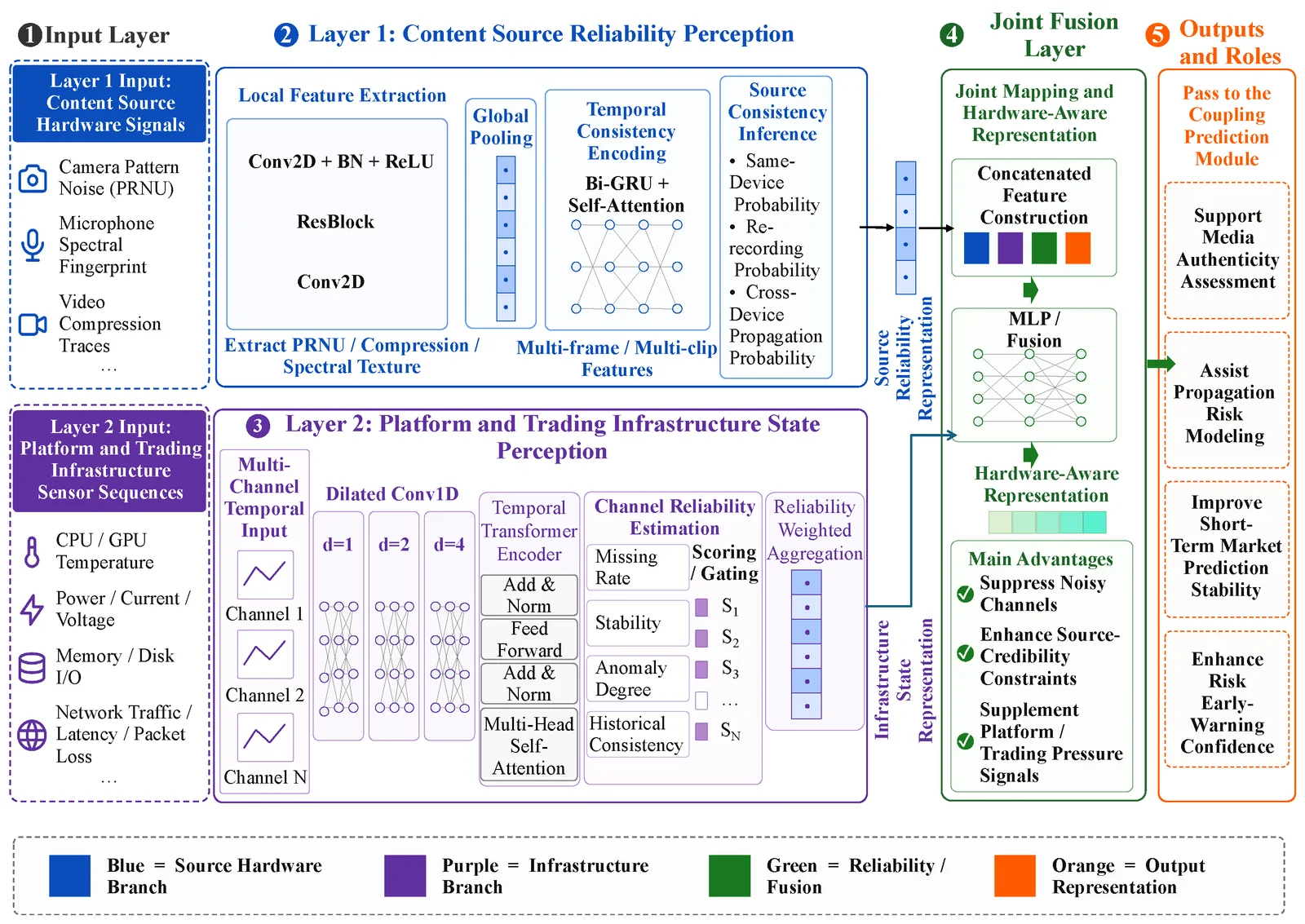
Architecture of the dual-layer hardware reliability perception and temporal encoding module.

The dual-layer hardware reliability perception and temporal encoding module is designed to extract both physical provenance evidence from content-acquisition devices and operating states from media platforms, market-data nodes, and trading infrastructures. As shown in Figure 2, let the hardware input associated with content provenance be denoted by $X^{s}\in R^{T_{s}\times H_{s}\times W_{s}\times C_{s}}$, where $T_{s}$ represents the number of consecutive frames or temporal segments, and $H_{s}$, $W_{s}$, and $C_{s}$ represent spatial height, spatial width, and channel number, respectively. The infrastructure sensing sequence is represented as $X^{i}\in R^{T_{i}\times C_{i}}$, where $C_{i}$ corresponds to channels such as temperature, power consumption, resource utilization, network traffic, latency, and packet loss. The source reliability perception branch adopts a hierarchical architecture consisting of $L_{s}$ residual convolutional stages and $L_{r}$ temporal encoding layers. At the *l*-th convolutional stage, a kernel of size $K_{l}^{h}\times K_{l}^{w}$ is used to map the input from $H_{l-1}\times W_{l-1}\times C_{l-1}$ to $H_{l}\times W_{l}\times C_{l}$, while spatial reduction is controlled by stride $S_{l}$. The corresponding feature transformation is expressed as

(16) $$U_{l}=\rho \left(N_{l}\left(C_{l}\left(U_{l-1};K_{l}^{h},K_{l}^{w},C_{l}\right)\right)\right)+P_{l}\left(U_{l-1}\right),$$

where $C_{l}$ denotes convolutional mapping, $N_{l}$ denotes normalization, ρ(·) denotes nonlinear activation, and $P_{l}$ aligns the spatial dimensions and channel width of the residual branch. Sensor pattern noise, local compression residuals, and microphone spectral textures are primarily extracted by shallow layers, whereas cross-frame device fingerprints, screen-recapture boundaries, and re-encoding traces are learned by deeper layers. Spatial features from each temporal segment are transformed into a sequence $Q^{s}\in R^{T_{s}\times D_{s}}$ through global statistical pooling and are subsequently processed by a bidirectional temporal encoder to jointly analyze visual motion, terminal posture, and acoustic variation:

(17) $${\vec{h}}_{t}=F_{f}\left(Q_{t}^{s},{\vec{h}}_{t-1}\right),\;{\overset{\leftarrow }{h}}_{t}=F_{b}\left(Q_{t}^{s},{\overset{\leftarrow }{h}}_{t+1}\right),\;h_{t}^{s}=\left[{\vec{h}}_{t};{\overset{\leftarrow }{h}}_{t}\right].$$

To identify physical consistency among heterogeneous hardware signals, temporal synchronization relationships among video motion vectors, terminal acceleration, angular velocity, acoustic-environment variations, and compression parameters are compared. A source-consistency representation is then constructed as

(18) $$r^{s}=\psi_{s}\left(\sum_{t=1}^{T_{s}}\alpha_{t}^{s}h_{t}^{s},\Delta_{\mathrm{motion}},\Delta_{\mathrm{audio}},\Delta_{\mathrm{compression}}\right),$$

where $\alpha_{t}^{s}$ denotes temporal importance, while $\Delta_{\mathrm{motion}}$, $\Delta_{\mathrm{audio}}$, and $\Delta_{\mathrm{compression}}$ quantify discrepancies in terminal movement, acoustic fingerprints, and compression trajectories, respectively. This representation is used to estimate same-device acquisition probability, secondary-recording probability, re-dubbing probability, and cross-device dissemination probability, but does not directly replace multimedia semantic authenticity assessment. The infrastructure state branch consists of $L_{c}$ dilated temporal convolutional layers and an $L_{i}$-layer long-range dependency encoder. At the *l*-th dilated convolutional layer, the kernel width is $K_{l}$, the number of channels is $C_{l}^{i}$, and the dilation factor is $d_{l}$. The corresponding output is given by

(19) $$V_{t,l}=\rho \left(\sum_{k=0}^{K_{l}-1}W_{k,l}V_{t-d_{l}k,l-1}+b_{l}\right).$$

The dilated structure enlarges the temporal receptive field without substantially increasing the number of parameters, allowing local network jitter, transient power fluctuations, and longer-term access pressure to be preserved simultaneously. The convolutional output is further processed by a temporal state encoder to obtain the infrastructure operating representation:

(20) $$Z^{i}=T_{i}\left(V_{1:T_{i},L_{c}}+E^{\mathrm{time}}+E^{\mathrm{channel}}\right),$$

where $E^{\mathrm{time}}$ represents the actual sampling interval and $E^{\mathrm{channel}}$ distinguishes heterogeneous sensing channels. To mitigate the effects of channel missingness, drift, and device failure, a reliability score is computed for the *c*-th channel according to its missing rate $m_{c}$, local fluctuation $s_{c}$, drift degree $d_{c}$, and historical consistency $h_{c}$:

(21) $$\eta_{c}=\exp\left(-\lambda_{m}m_{c}-\lambda_{s}s_{c}-\lambda_{d}d_{c}+\lambda_{h}h_{c}\right),\;{\tilde{\eta }}_{c}=\frac{\eta_{c}}{\varepsilon +\sum_{j=1}^{C_{i}}\eta_{j}}.$$

The infrastructure pressure representation is obtained by reliability-weighted aggregation of channel-specific states:

(22) $$r^{i}=\sum_{c=1}^{C_{i}}{\tilde{\eta }}_{c}G_{c}\left(Z_{:,c}^{i}\right).$$

The proposed design provides a provable robustness property against sensing noise. Suppose that the *c*-th channel is affected by a disturbance $\delta_{c}$, and that the encoding function $G_{c}$ satisfies the Lipschitz condition

(23) $$\left|G_{c}\left(x+\delta_{c}\right)-G_{c}\left(x\right)\right|\leq L_{c}\left|\delta_{c}\right|.$$

Because the reliability statistics are recomputed from the perturbed input, the weights are not fixed under disturbance. Let $\tilde{\eta }\left(X\right)$ denote the normalized reliability-weight vector, assume it is locally $K_{\eta }$-Lipschitz, and let $B_{G}$ bound the encoded channel norms. Adding and subtracting the perturbed features under the original weights gives the following dynamic-weight bound:

(24) $$\begin{array}{cc} ∥r^{i}\left(X+\delta \right)-r^{i}\left(X\right)∥\; & \leq \sum_{c=1}^{C_{i}}{\tilde{\eta }}_{c}\left(X+\delta \right)L_{c}∥\delta_{c}∥+B_{G}{∥\tilde{\eta }\left(X+\delta \right)-\tilde{\eta }\left(X\right)∥}_{1}\\ \; & \leq \sum_{c=1}^{C_{i}}{\tilde{\eta }}_{c}\left(X+\delta \right)L_{c}∥\delta_{c}∥+B_{G}K_{\eta }∥\delta ∥. \end{array}$$

The first term captures changes in the encoded sensing values and the second captures the simultaneous change in the reliability weights. Thus, Equation (24) no longer treats the weights as constants. A channel with increased missingness, fluctuation, or drift receives a smaller perturbed weight ${\tilde{\eta }}_{c}\left(X+\delta \right)$, while the $K_{\eta }$ term makes explicit the additional sensitivity introduced by adapting the weights. In implementation, missingness is computed over the current causal observation window, stability is the median absolute first difference normalized by the training-set interquartile range, drift is the population-stability index against the training reference, and historical consistency is the exponentially weighted correlation with redundant channels. Finally, the source reliability representation $r^{s}$ and the infrastructure pressure representation $r^{i}$ are jointly projected to form the hardware-aware representation

(25) $$F^{h}=\phi_{h}\left([r^{s};r^{i};r^{s}⊙r^{i};\left|r^{s}-r^{i}\right|]\right),$$

which is delivered to the media propagation–market response coupling prediction module. Physical provenance constraints are thereby provided for multimedia risk semantics, while real-time states such as platform access pressure, market-data request growth, and trading-network congestion are simultaneously incorporated. High-sentiment information originating from suspicious sources, credible information with limited propagation, and abrupt events accompanied by synchronous increases in media traffic and trading infrastructure pressure can, therefore, be distinguished. Improved stability is consequently achieved for short-term market prediction, risk-level identification, and early warning under modality missingness, sensing noise, and device-state variation.

#### 3.3.4. Media Propagation-Market Response Coupling Prediction Module

**Figure 3:**
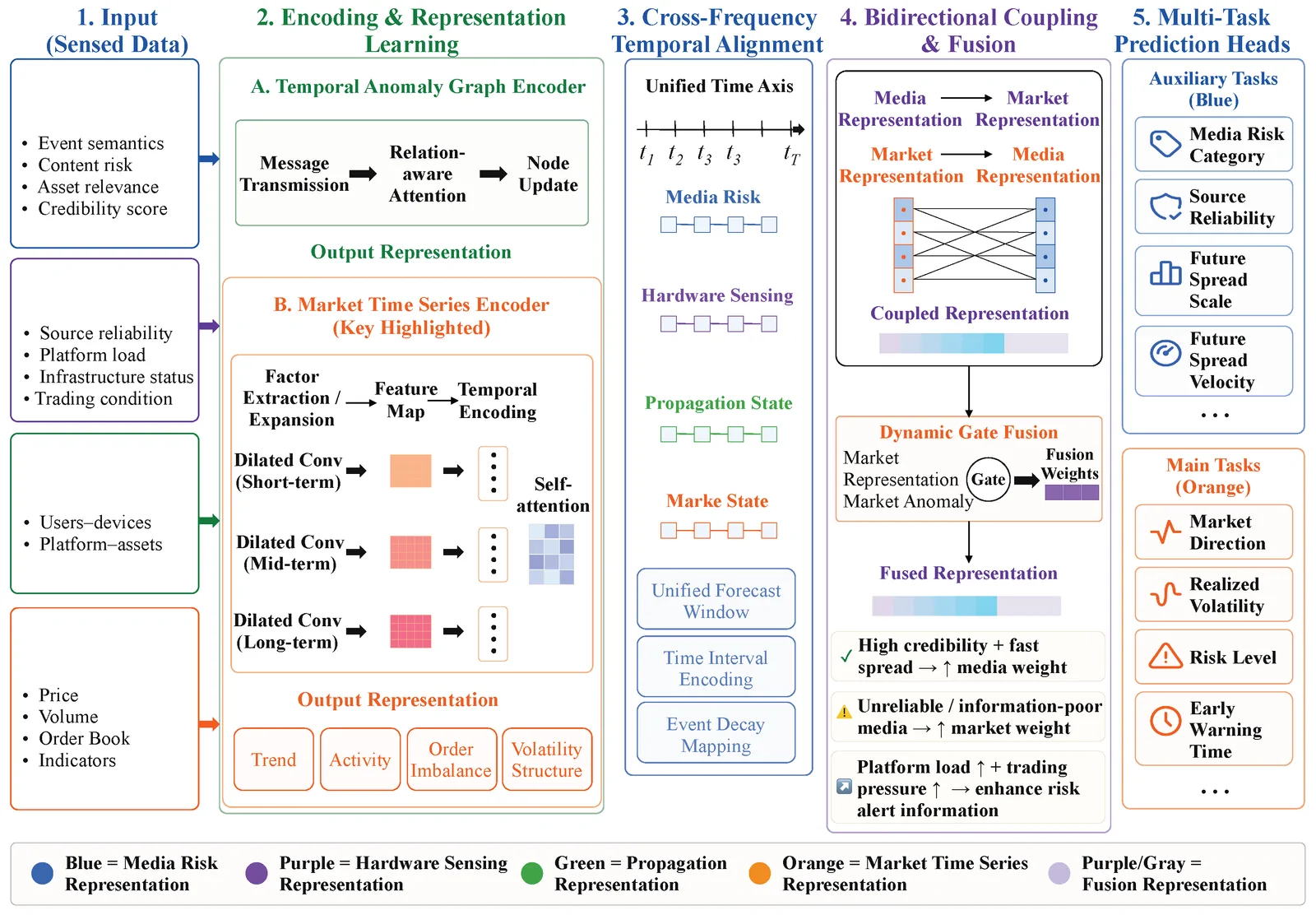
Architecture of the media propagation–market response coupling prediction module.

The media propagation–market response coupling prediction module receives the integrated media risk representation $F^{r}$, the hardware-aware representation $F^{h}$, the heterogeneous propagation graph $G_{t}$, and the historical market sequence $X^{m}\in R^{T_{m}\times C_{m}}$. The dynamic process through which risk information evolves from content generation and user diffusion to platform migration and asset-price response is thereby modeled. As shown in Figure 3, the propagation graph contains $N_{g}$ nodes, $R_{g}$ relation types, and an adjacency tensor $A\in R^{N_{g}\times N_{g}\times R_{g}}$. Initial node representations derived from media semantics, user behavior, device provenance, platform states, and asset attributes are projected into a shared space with width $D_{g}^{\left(0\right)}$. The graph encoder consists of $L_{g}$ relation-aware propagation layers. At the *l*-th layer, $M_{l}$ relation-attention heads are used, the output width is $D_{g}^{\left(l\right)}$, and independent projection parameters are assigned to heterogeneous relations. The state of node *v* at layer l+1 is defined as

(26) $$h_{v}^{\left(l+1\right)}=\varphi \left(W_{s}^{\left(l\right)}h_{v}^{\left(l\right)}+\sum_{r=1}^{R_{g}}\sum_{u\in N_{r}\left(v\right)}\frac{\omega_{uvr}^{\left(l\right)}}{\left|N_{r}\left(v\right)\right|}W_{r}^{\left(l\right)}h_{u}^{\left(l\right)}\right).$$

The attention weight $\omega_{uvr}^{\left(l\right)}$ is determined jointly by content risk, source reliability, propagation time difference, and asset relevance:

(27) $$\omega_{uvr}^{\left(l\right)}=\frac{\exp\left(q_{r}^{(l)T}\tanh\left(U_{r}^{\left(l\right)}\left[h_{u}^{\left(l\right)};h_{v}^{\left(l\right)};F_{u}^{r};F_{u}^{h};\Delta t_{uv}\right]\right)\right)}{\sum_{j\in N_{r}\left(v\right)}\exp\left(q_{r}^{(l)T}\tanh\left(U_{r}^{\left(l\right)}\left[h_{j}^{\left(l\right)};h_{v}^{\left(l\right)};F_{j}^{r};F_{j}^{h};\Delta t_{jv}\right]\right)\right)}.$$

Greater contributions are consequently assigned to nodes with higher credibility, stronger asset relevance, and shorter propagation intervals, whereas the effects of abnormal sources or semantically conflicting content are suppressed during propagation aggregation. After $L_{g}$ graph updates, event-node-guided and asset-node-guided dual readout is performed to obtain the propagation representation $F^{p}$, which encodes the current diffusion scale, propagation velocity, cross-platform migration intensity, and critical propagation paths. The market branch consists of $L_{m}$ causal temporal blocks. Each block contains a depthwise separable convolution with kernel width $K_{l}$ and channel number $C_{l}^{m}$, a temporal projection layer with width $D_{m}^{\left(l\right)}$, and a causal self-attention layer. The market state is updated as

(28) $$Y_{t}^{\left(l\right)}=\chi_{l}\left(\sum_{k=0}^{K_{l}-1}D_{k}^{\left(l\right)}⊙Y_{t-k}^{\left(l-1\right)}\right)+B_{l}Y_{t}^{\left(l-1\right)}.$$

The causal index t−k≤t guarantees that observations occurring after the prediction time are excluded from the encoding process. A market representation $F^{m}$ containing price trends, trading activity, order-book imbalance, and short-term volatility structures is consequently obtained. To unify discrete media events, minute-level propagation states, second-level hardware states, and high-frequency market sequences, all branch representations are projected onto a shared temporal grid of size $T_{c}\times D_{c}$ ending at the forecast origin $t_{0}$, i.e., $\tau \in [t_{0}-W,t_{0}]$. Asynchronous observations are distributed using a strictly causal, one-sided continuous-time decay kernel:

(29) $$\begin{array}{cc} {\tilde{F}}_{\tau }^{q}\; & =\frac{\sum_{j}\kappa_{q}\left(\tau -t_{j}\right)F_{j}^{q}}{\epsilon +\sum_{j}\kappa_{q}\left(\tau -t_{j}\right)},\\ \kappa_{q}\left(\tau -t_{j}\right)\; & =1\left\{t_{j}\leq \tau \leq t_{0}\right\}\exp\left(-\beta_{q}{\left(\tau -t_{j}\right)}^{\gamma_{q}}\right),\;q\in r,h,p,m. \end{array}$$

The indicator is evaluated before smoothing, so an observation with $t_{j}>\tau$ has exactly zero weight; no centered padding, interpolation across $t_{0}$, or backward fill is permitted. Target intervals $\left[t_{0},t_{0}+h\right]$ are constructed only after the input tensor has been frozen. Therefore, for any horizon h∈{5,15,30,60} min, every input is measurable with respect to the information set available at $t_{0}$, and neither the prediction interval nor later observations can enter the aligned representation. Bidirectional coupling is subsequently established between media propagation and market states. The media-to-market branch generates a market-impact representation from propagation and risk features, whereas the market-to-media branch estimates the feedback of price, trading-volume, and order-book changes on subsequent discussion and diffusion:

(30) $$C_{p\to m}=\tanh\left({\tilde{F}}^{p}W_{pm}{\tilde{F}}^{mT}\right){\tilde{F}}^{m},\;C_{m\to p}=\tanh\left({\tilde{F}}^{m}W_{mp}{\tilde{F}}^{pT}\right){\tilde{F}}^{p}.$$

The four aligned representations and the bidirectional coupling representations are then processed by a gated fusion network. The two quantities used in the fusion equation are defined explicitly as

$$\begin{array}{cc} F^{\mathrm{media}} & =\phi_{\mathrm{media}}\left(\left[{\tilde{F}}^{r};{\tilde{F}}^{h};{\tilde{F}}^{p};C_{p\to m};C_{m\to p}\right]\right),\\ F^{\mathrm{market}} & =\phi_{\mathrm{market}}\left(\left[{\tilde{F}}^{m};{\tilde{F}}^{h}\right]\right). \end{array}$$

Here, $\phi_{\mathrm{media}}$ and $\phi_{\mathrm{market}}$ are two-layer projections to the common fusion dimension. Thus, $F^{\mathrm{media}}$ is the reliability-conditioned content/propagation state, whereas $F^{\mathrm{market}}$ is the causal price/liquidity state conditioned on contemporaneously available infrastructure telemetry. The gate is generated from media credibility, propagation intensity, hardware reliability, and market abnormality:

(31) $$z=\sigma \left(W_{z}\left[{\tilde{F}}^{r};{\tilde{F}}^{h};C_{p\to m};C_{m\to p};{\tilde{F}}^{m}\right]+b_{z}\right),\;F^{c}=z⊙F^{\mathrm{media}}+\left(1-z\right)⊙F^{\mathrm{market}}.$$

When content is credible, rapidly propagated, and strongly associated with the target asset, *z* increases the contribution of the media propagation branch. When media observations are sparse, sources are suspicious, or the graph structure is incomplete, greater importance is assigned to market sequences and hardware operating states. The fused representation $F^{c}$ is subsequently delivered to the direction classification head, volatility regression head, risk-level classification head, and propagation prediction head. Market direction, realized volatility, risk level, propagation scale, and propagation velocity are thereby predicted over multiple forecasting horizons. The proposed graph module is permutation equivariant. Let *P* denote an arbitrary node permutation matrix. For the permuted graph representation $H^{\prime }=PH$ and adjacency matrices $A_{r}^{\prime }=PA_{r}P^{T}$, relation-aware aggregation satisfies

(32) $$F_{g}\left(PH,{\left\{PA_{r}P^{T}\right\}}_{r=1}^{R_{g}}\right)=PF_{g}\left(H,{\left\{A_{r}\right\}}_{r=1}^{R_{g}}\right).$$

The model output, therefore, does not depend on the storage order of user, device, or content nodes and is determined only by the actual propagation relationships. Furthermore, since 0≤z≤1, if the media and market branches are affected by disturbances $\delta_{p}$ and $\delta_{m}$, respectively, the fusion error satisfies

(33) $$\left|\Delta F^{c}\right|\leq {\left|z\right|}_{\infty }\left|\delta_{p}\right|+{\left|1-z\right|}_{\infty }\left|\delta_{m}\right|.$$

This inequality indicates that the gated mechanism constrains the amplification of abnormalities from an individual branch. Content authenticity, propagation dynamics, hardware operating pressure, and endogenous market states are, therefore, incorporated into a unified dynamic framework. Information with extensive propagation but weak asset relevance can be distinguished from abrupt events characterized by credible risk content and synchronized increases in platform traffic and trading pressure, thereby improving the accuracy and stability of short-term market prediction and early risk warning.

## 4. Results and Discussion

### 4.1. Experimental Configuration

#### 4.1.1. Hardware and Software Platform

All experiments were conducted on a server equipped with two Intel Xeon Gold 6430 processors, 512 GB of DDR5 memory, and four NVIDIA A100 GPUs with 80 GB of memory each. NVMe solid-state drives were used to store multimedia content, propagation graphs, and high-frequency market sequences. High-speed inter-GPU data exchange was enabled through NVLink to support the parallel training of text, image, audio, video, hardware sensing sequences, and dynamic graph data. Hardware data related to content provenance were primarily collected using cameras, microphones, and mobile-terminal sensors. Media-platform and trading-infrastructure data included CPU and GPU temperatures, device power consumption, memory utilization, disk input/output, network latency, packet loss, and port traffic. All baseline models were evaluated under the same hardware environment to minimize the influence of computational-platform differences on training time and predictive performance. Ubuntu 22.04 LTS was adopted as the operating system. Deep-learning models were implemented using Python 3.10 and PyTorch 2.2, with GPU acceleration provided by CUDA 12.1 and cuDNN 8.9. Text preprocessing and FinBERT inference were implemented using Transformers 4.39. Image and video processing was performed using OpenCV and Torchvision, whereas audio processing was conducted using Librosa and Torchaudio. Propagation graphs and heterogeneous dynamic graphs were modeled using PyTorch Geometric and DGL. Pandas, NumPy, and SciPy were employed for the cleaning and statistical analysis of market sequences and hardware sensing data. XGBoost 2.0 was used to implement the XGBoost baseline, and evaluation metrics were calculated using Scikit-learn. TensorBoard was employed to record training losses, validation performance, and learning-rate variations. The random seeds of Python, NumPy, and PyTorch were fixed to improve experimental reproducibility. For the language branch, Transformers revision 4.39 loaded the public ProsusAI/finbert checkpoint with its original WordPiece vocabulary. The entire encoder and the six semantic heads were fine-tuned end to end; therefore, “FinBERT” in the baseline denotes an independently fine-tuned text-only model, while the FMRP-Net branch denotes the same initialization coupled to the other sensing modules. Reproducibility materials specify the checkpoint hash, maximum length, optimizer groups, label maps, seeds, and fine-tuning script. The dataset was divided chronologically according to event occurrence time, with 70%, 15%, and 15% of the samples assigned to the training, validation, and test sets, respectively. The same event and its derivative content were prevented from appearing in different subsets, thereby avoiding event leakage and future-information leakage. Five-fold cross-validation was further performed within the training set. In each fold, four subsets were used for model training and the remaining subset was used for validation, and the final performance was calculated as the average over the five runs. Except for XGBoost, all deep-learning models were optimized using AdamW. The initial learning rate was set to $1\times 10^{-4}$, the weight-decay coefficient was set to $1\times 10^{-5}$, the batch size was set to 32, and the maximum number of training epochs was set to 100. Early stopping was activated when validation performance failed to improve for 10 consecutive epochs. The learning-rate warm-up ratio was set to 10%, after which cosine annealing was applied. The gradient-clipping threshold was set to 1.0. The hidden dimension was uniformly set to 256, with 4 Transformer encoding layers, 8 attention heads, a feed-forward dimension of 1024, and a dropout rate of 0.2. The maximum text length was set to 256 tokens, while the input windows for market sequences and hardware sensing sequences were set to the preceding 120 time steps. The node representation dimension of the dynamic graph model was set to 256. In the multi-task objective, the initial weights for market direction classification, volatility regression, risk-level classification, and propagation-trend prediction were all set to 1.0. The final hyperparameter settings were determined according to the combined Macro-F1 and RMSE performance on the validation set. All scalers, missingness references, drift baselines, vocabulary-dependent preprocessing, and decision thresholds were fitted on the training data only. For each test event, a hard cutoff was applied at $t_{0}$ before resampling; feature joins required source timestamps $t_{j}\leq t_{0}$, and the causal kernel in Equation (29) was then applied. A unit test shifted every post-$t_{0}$ value by a random offset and verified bitwise-identical input tensors and predictions, providing an implementation-level check against forecast-window leakage.

#### 4.1.2. Threshold Selection and Operational Definitions

All operational thresholds were selected once on the chronological validation set and then frozen. Candidate probability thresholds from 0.30 to 0.90 in increments of 0.05 were evaluated. The media-risk threshold (0.70) maximized validation Macro-F1; the source-reliability threshold (0.65) maximized Youden’s *J* for original/same-device acquisition versus screen recapture, re-dubbing, cross-device recording, and severe recompression; and the cross-modal consistency threshold (0.55) maximized balanced accuracy. A source is operationally “reliable” only when its calibrated source-reliability probability is at least 0.65 and no hard conflict is triggered by a device-timestamp mismatch or an asset-association score below 0.60. These rules use learned provenance and manipulation evidence, not account identity alone.

An early warning is the first time $t_{\mathrm{alarm}}\leq t_{0}$ at which the high-risk probability is at least 0.70 for two consecutive one-minute updates. Realized risk onset $t_{\mathrm{onset}}$ is the earliest subsequent time at which either five-minute realized volatility exceeds the 95th percentile estimated from the training period or the absolute mid-price return exceeds two training-period standard deviations. Lead time is $t_{\mathrm{onset}}-t_{\mathrm{alarm}}$ for correctly warned events; false alarms are counted separately in the classification metrics and do not inflate lead time. Kernel half-lives, network widths, dropout, and task weights were chosen by the preregistered validation objective Macro-F1−5RMSE, with no test-set adjustment.

#### 4.1.3. Baseline Models and Evaluation Metrics

The selected baseline models included XGBoost [47], LSTM [48], Informer [49], FinBERT [46], Multimedia Transformer [50], DeepHawkes [51], Temporal Graph Network [52], Text–Market Fusion Transformer [53], and Sensor–Market Fusion Transformer [54]. Three recent competitors were additionally reproduced: PatchTST [55], TimesNet [56], and the finance-specific Modality-aware Transformer (MAT) [57]. PatchTST tests whether patch-wise channel-independent temporal modeling is sufficient; TimesNet tests multi-period two-dimensional temporal variation modeling; and MAT provides a modern multimodal financial Transformer with intra-, inter-, and target-modality attention. Official implementations were adapted only at their input/output layers, and all models received the same causal observation windows, chronological splits, tuning budget, and prediction heads. XGBoost learns nonlinear relationships between market indicators and prediction labels by integrating multiple gradient-boosted decision trees, offering high training efficiency and strong suitability for structured data. LSTM controls the transmission of historical information through input, forget, and output gates, enabling long-term dependencies in time series to be captured. Informer processes long sequences using probabilistic sparse self-attention and sequence distillation, thereby reducing the computational cost associated with high-frequency market data. FinBERT is pretrained on corpora and extracts sentiment and event semantics from text, providing strong representation capability for domain-specific expressions. Multimedia Transformer integrates textual, visual, audio, and video features through cross-attention, allowing complex dependencies among heterogeneous media modalities to be modeled. DeepHawkes combines recurrent neural networks with Hawkes processes to predict information diffusion and jointly characterize propagation structures and temporal dynamics. Temporal Graph Network updates dynamic graph states through temporal encoding, message passing, and node memory, making it suitable for continuously evolving user propagation relationships. Text–Market Fusion Transformer integrates text and market sequences through cross-attention, enabling sentiment semantics and price variations to be jointly exploited. Sensor–Market Fusion Transformer combines sensing signals, including server power consumption, temperature, and network latency, with market data, thereby supplementing real-time market pressure information from infrastructure operating states. Accuracy, Macro-F1, ROC-AUC, matthews correlation coefficient (MCC), PR-AUC, mean absolute error (MAE), root mean squared error (RMSE), mean absolute percentage error (MAPE), and $R^{2}$ were used to evaluate model performance. To reduce space devoted to standard textbook material, the generic metric formulas have been removed. Macro-F1 is the unweighted mean of class-wise F1 scores; ROC-AUC and PR-AUC are computed from continuous scores; MCC uses the standard confusion-matrix definition; and MAE, RMSE, MAPE, $R^{2}$, and Spearman correlation use their conventional sample definitions. Confidence intervals are percentile intervals from 1000 event-cluster bootstrap resamples, preserving all horizons and derivative records of a root event within the same resample.

### 4.2. Short-Term Market Direction Prediction

This experiment was designed to evaluate the overall capabilities of different models in predicting market directions over future horizons of 5, 15, 30, and 60 min. Particular emphasis was placed on assessing the incremental contributions of structured market variables, temporal dependencies, semantics, multimedia content, propagation relationships, and hardware sensing states to prediction performance.

**Figure 4:**
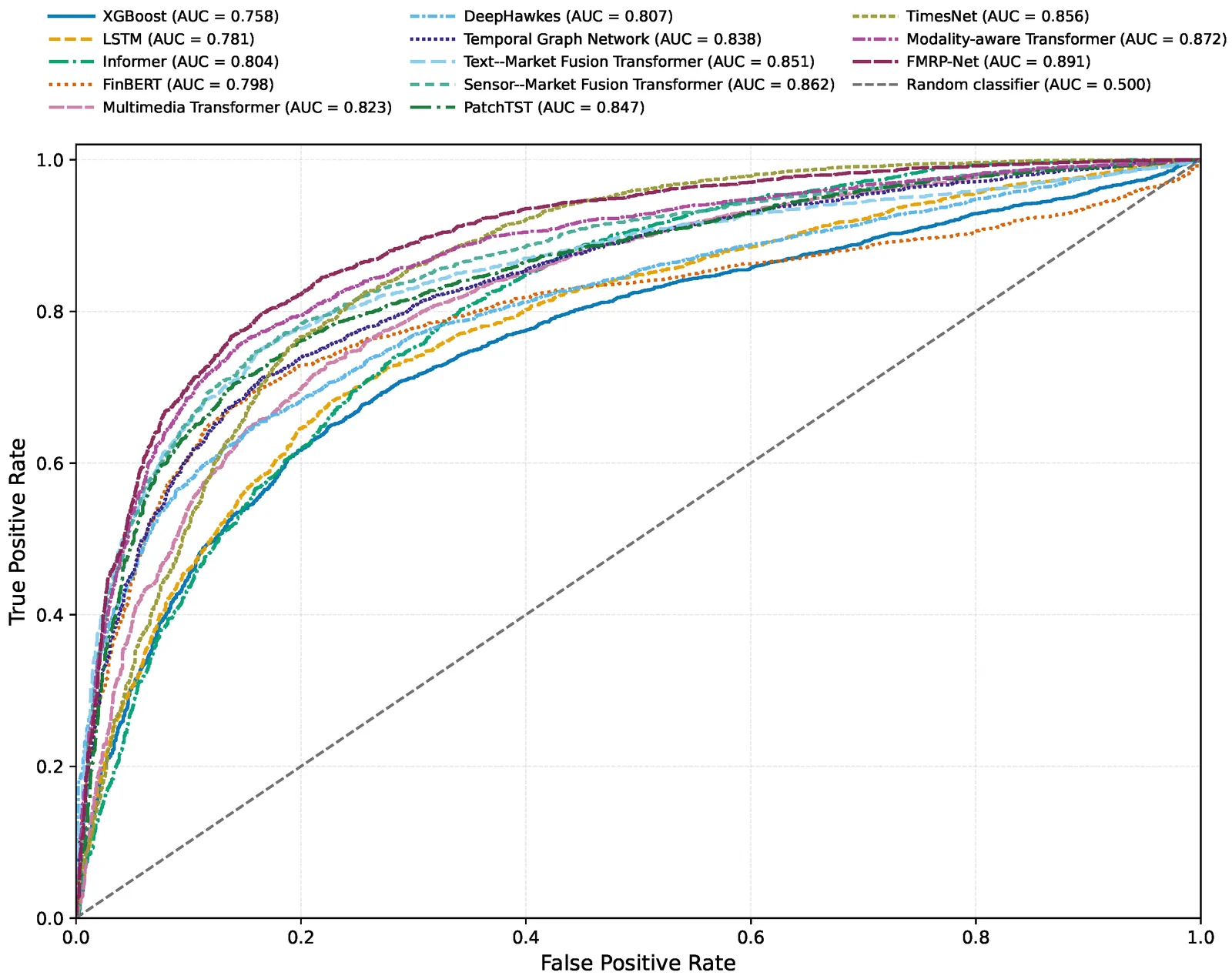
ROC curve comparison of different methods for short-term market direction prediction.

As shown in Table 2 and Figure 4, XGBoost achieved an Accuracy of 0.704 and a Macro-F1 of 0.691, representing the weakest overall performance among the evaluated methods. This result indicates that the continuous evolution of market states cannot be adequately characterized solely by partitioning structured feature spaces with tree-based models. The Accuracy obtained by LSTM increased to 0.731, demonstrating that gated memory mechanisms can capture temporal dependencies embedded in price and trading-volume sequences. Informer further achieved an Accuracy of 0.752 and a ROC-AUC of 0.804, suggesting that informative temporal positions can be identified effectively from long sequences through sparse attention. FinBERT achieved an Accuracy of 0.742, outperforming XGBoost and LSTM but remaining inferior to Informer. Semantics were, therefore, shown to provide useful predictive information, although endogenous market dynamics could not be fully represented using textual content alone. Multimedia Transformer achieved an Accuracy of 0.768 and a ROC-AUC of 0.823, confirming that joint representations of images, audio, video, and text provide complementary information beyond that available from a single textual modality. DeepHawkes achieved a ROC-AUC of 0.807 but an Accuracy of only 0.746. Although diffusion intensity could be identified through the self-exciting propagation mechanism, market price states were not described sufficiently. The Accuracy of Temporal Graph Network reached 0.781 through dynamic node-state updating, indicating that propagation structures and user relationships provide meaningful explanatory information for short-term market direction. Text–Market Fusion Transformer and Sensor–Market Fusion Transformer achieved Accuracy values of 0.794 and 0.803, respectively. The latter was the strongest of the original baseline set, demonstrating that infrastructure operating states can provide more immediate market-pressure information than textual signals alone. FMRP-Net achieved the best results, with an Accuracy of 0.832, a Macro-F1 of 0.824, a ROC-AUC of 0.891, an MCC of 0.665, and a PR-AUC of 0.883. The narrowest confidence intervals were also obtained, indicating superior predictive accuracy and greater stability across repeated time-series experiments. Among the added modern competitors, PatchTST, TimesNet, and MAT achieved ROC-AUC values of 0.847, 0.856, and 0.872, respectively. MAT was the strongest baseline after explicitly modeling textual and numerical modalities, but FMRP-Net retained gains of 2.0 percentage points in Accuracy and 1.9 points in ROC-AUC. These gains are consistent with the contribution of provenance, propagation topology, infrastructure pressure, and reliability adaptation beyond a modern multimodal attention backbone.

From the perspective of model mechanisms, nonlinear relationships are approximated by XGBoost through piecewise constant functions. However, explicit temporal states are absent, making path dependence and state transitions in high-frequency markets difficult to represent. Historical information is accumulated recursively by LSTM through gated transitions, but long-range information may gradually decay during sequential computation, and asynchronous signals from multiple sources are difficult to model simultaneously. Long-distance temporal relationships are established directly by Informer through attention weights, explaining its advantage over recurrent models, although its inputs remain predominantly concentrated on market sequences. The pretrained semantic space of FinBERT enables events and sentiment orientations to be identified, but textual sentiment does not correspond directly to actual market impact. Its performance is, therefore, constrained by source distortion, asset mismatch, and delayed market reactions. Cross-modal attention is employed by Multimedia Transformer to learn correlations among heterogeneous modalities, but information authenticity and propagation processes are not explicitly constrained. The conditional-intensity formulation of DeepHawkes is well suited to modeling the self-exciting characteristics of reposting events, although market responses are simplified as external consequences of propagation. Temporal Graph Network aggregates user, content, and temporal-neighborhood information through message passing, allowing changes in propagation topology to be represented. Nevertheless, fine-grained market sequences and hardware operating evidence are not incorporated. The two fusion Transformers alleviate the limitations of single-source inputs through joint attention. Sensor–Market Fusion Transformer performs better because real-time variables such as power consumption, latency, and traffic provide timely indications of market pressure. In FMRP-Net, distorted content is suppressed through cross-modal consistency assessment, abnormal sensing channels are downweighted through reliability-aware weighting, and the effects of media propagation on market states and the feedback of market conditions on subsequent discussions are jointly characterized through heterogeneous dynamic graphs, cross-frequency alignment, and bidirectional coupling. More balanced class recognition, stronger discrimination between positive and negative samples, and greater stability across repeated experiments are, therefore, achieved.

### 4.3. Future Market Volatility Prediction

This experiment was designed to evaluate the ability of different models to predict market volatility over future horizons of 5, 15, 30, and 60 min. The incremental contributions of market sequences, semantics, multimedia information, propagation relationships, and hardware sensing states to continuous risk characterization were also investigated.

**Figure 5:**
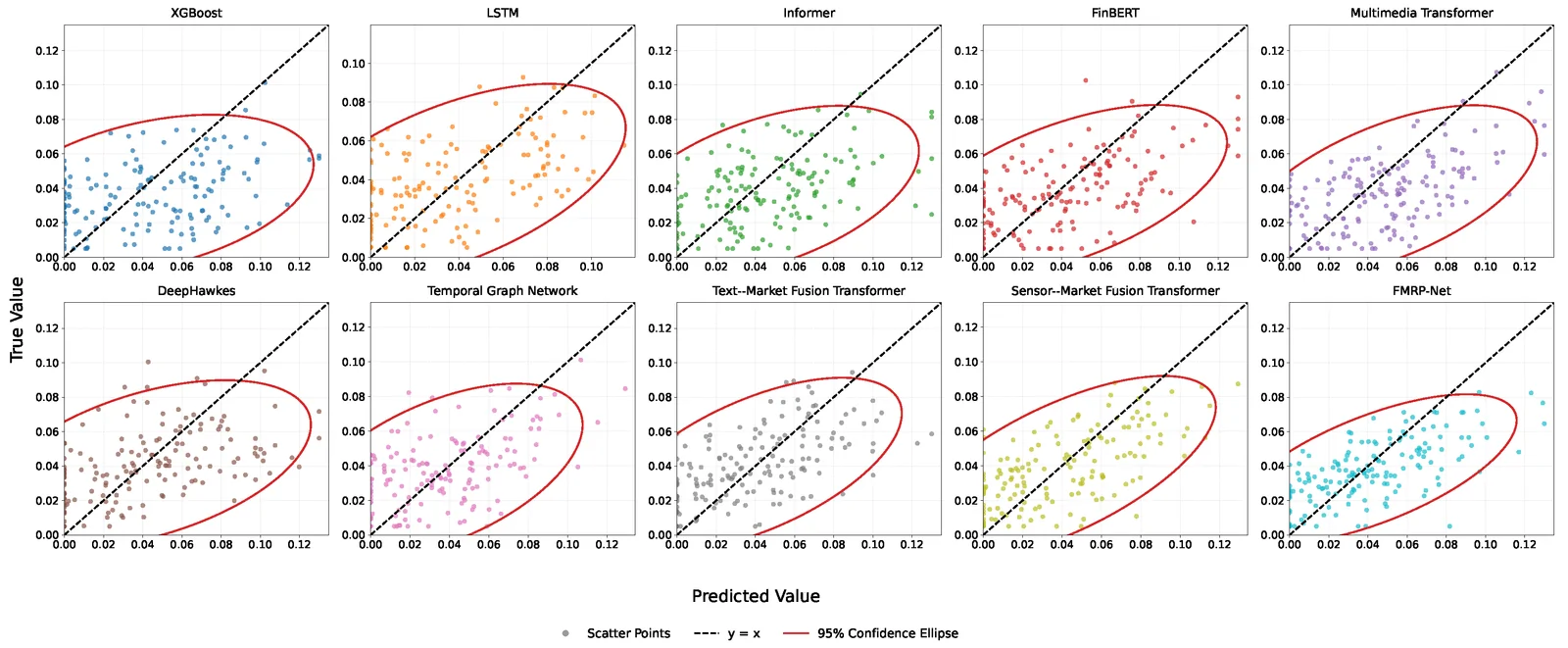
Comparison of the distributions of predicted and ground-truth values for market volatility prediction, where the dashed line denotes ideal agreement and the ellipses represent the 95% confidence regions.

As shown in Table 3 and Figure 5, XGBoost produced an MAE of 0.0268, an RMSE of 0.0389, and a MAPE of 18.73%, resulting in the largest overall prediction errors. This finding indicates that continuous volatility dynamics are difficult to describe using a static tree-based model. The MAE was reduced to 0.0243 by LSTM, demonstrating that recurrent memory can capture temporal dependencies in market sequences. Informer achieved an MAE of 0.0229 and an $R^{2}$ of 0.691, indicating that long-sequence attention improves the modeling of medium- and long-range volatility patterns. FinBERT achieved an MAE of 0.0251, outperforming XGBoost but remaining inferior to the principal temporal models. Although textual semantics can reflect market risk expectations, the underlying price-volatility process cannot be reconstructed independently from textual information. The MAE was further reduced to 0.0218 by Multimedia Transformer, confirming that images, audio, video, and text provide more comprehensive information concerning event-related shocks. DeepHawkes achieved an MAE of 0.0235, indicating that self-exciting propagation intensity contains useful predictive information, although endogenous market states remain insufficiently characterized. Temporal Graph Network achieved an MAE of 0.0211 and an $R^{2}$ of 0.739, demonstrating a strong association between evolving propagation structures and volatility changes. Text–Market Fusion Transformer and Sensor–Market Fusion Transformer achieved MAE values of 0.0202 and 0.0196, respectively. The latter was the strongest of the original baseline set, indicating that infrastructure operating states provide timely information concerning market pressure. FMRP-Net achieved the best performance, with an MAE of 0.0178, an RMSE of 0.0271, a MAPE of 12.46%, an $R^{2}$ of 0.812, and a Spearman correlation coefficient of 0.849. The narrowest confidence intervals were also observed, demonstrating lower prediction errors, stronger consistency in volatility ranking, and greater stability across repeated experiments. The modern baselines produced the same ordering in volatility prediction: MAT was strongest with an MAE of 0.0189, followed by TimesNet and PatchTST. FMRP-Net reduced MAE by 5.8% relative to MAT and improved $R^{2}$ from 0.786 to 0.812, while preserving the reported confidence-interval ordering.

From a modeling perspective, nonlinear mappings between input features and volatility are approximated by XGBoost through piecewise regions. However, because continuous state transitions are not explicitly represented, volatility clustering and long-memory effects may be overlooked. Historical information is retained by LSTM through gated state recursion, allowing volatility persistence to be described, but distant information may be lost through recurrent compression. Informative temporal positions are selected by Informer through sparse attention, expanding the effective receptive field and producing better performance than LSTM. Nevertheless, market history remains its principal source of predictive information. Semantic vectors generated by FinBERT capture risk terminology, event categories, and sentiment changes, but temporal delays and authenticity discrepancies remain between textual impact and realized volatility. Cross-modal correlation matrices are used by Multimedia Transformer to model interactions among heterogeneous media features, allowing visual and acoustic risk signals absent from text to be identified. Historical events are accumulated by DeepHawkes through conditional intensity, making the model suitable for sudden information diffusion, although order-book and price variations are not directly represented. User, content, and platform node states are updated by Temporal Graph Network through temporal message passing, thereby preserving the evolution of propagation topology. The two fusion Transformers reduce information insufficiency through joint representations. Sensor–Market Fusion Transformer achieves better performance because server load, network traffic, and latency are highly sensitive to real-time pressure. In FMRP-Net, distorted information is filtered through cross-modal consistency assessment, abnormal sensing channels are suppressed through dynamic reliability weighting, and external information shocks and endogenous market volatility are jointly modeled through cross-frequency alignment and bidirectional propagation–market coupling. Extreme prediction errors are consequently reduced, ranking consistency is improved, and future volatility changes are captured more accurately.

### 4.4. Additional Robustness, Stability, and Efficiency Analysis

#### 4.4.1. Horizon-Resolved Performance

Table 4 disaggregates the previously reported averages. Direction and volatility prediction become gradually more difficult as the horizon increases, as expected in an efficient market, but the degradation is smooth rather than abrupt. The arithmetic means of the four rows reproduce the aggregate Accuracy (0.832), Macro-F1 (0.824), ROC-AUC (0.891), MAE (0.0178), RMSE (0.0271), MAPE (12.46%), and $R^{2}$ (0.812) reported in Table 2 and Table 3.

#### 4.4.2. Device-Disjoint Source Reliability

To distinguish manipulation cues from memorization of known devices, the controlled provenance subset was repartitioned by physical device identity and model family. The subset contained 38 physical devices from 14 camera, phone, and microphone model families; no device and no model family in the test fold appeared in training or validation. Original recordings and each manipulation type were balanced within each fold. As shown in Table 5, the complete provenance branch retained an AUC of 0.897 on unseen device families, only 0.032 below the ordinary event split and well above a fingerprint-only classifier. The smaller gap in manipulation Macro-F1 and the substantially lower equal-error rate show that the model exploits recapture boundaries, re-encoding trajectories, audio re-dubbing, motion/acoustic inconsistency, and cross-modal evidence in addition to device fingerprints.

#### 4.4.3. Hyperparameter Sensitivity

The principal capacity, regularization, and temporal-alignment choices were varied one factor at a time around the validation-selected configuration. Table 6 shows that the selected hidden width of 256, dropout of 0.2, and alignment half-life of 15 min provide the best joint validation result, while neighboring settings change Macro-F1 by at most 0.009 and RMSE by at most 0.0006. The conclusions are, therefore, not tied to a narrow setting. Width 384 was not selected because its negligible accuracy change did not justify the additional memory and latency.

#### 4.4.4. Computational Cost

Table 7 reports end-to-end model cost on one NVIDIA A100 GPU using batch size 1 for inference and the same early-stopping rule for training. FMRP-Net is more expensive than the two strongest original fusion baselines because it includes multimedia encoders and a temporal propagation graph. Nevertheless, its 43.6 ms per-event latency remains well below the one-minute propagation update interval. Relative to Sensor–Market Fusion Transformer, the additional cost yields gains of 2.9 percentage points in Accuracy, 2.9 points in ROC-AUC, and a 9.2% reduction in volatility RMSE. For memory-constrained deployments, encoders can be cached per content item and graph updates can be performed incrementally.

### 4.5. Ablation Study

This experiment was designed to evaluate the practical contributions of the principal components of FMRP-Net by removing the pretrained financial-language event semantic branch, multimedia branches, cross-modal consistency mechanism, source hardware perception branch, infrastructure sensing branch, dynamic reliability weighting, temporal propagation graph, cross-frequency alignment, and multi-task learning mechanism individually.

**Figure 6:**
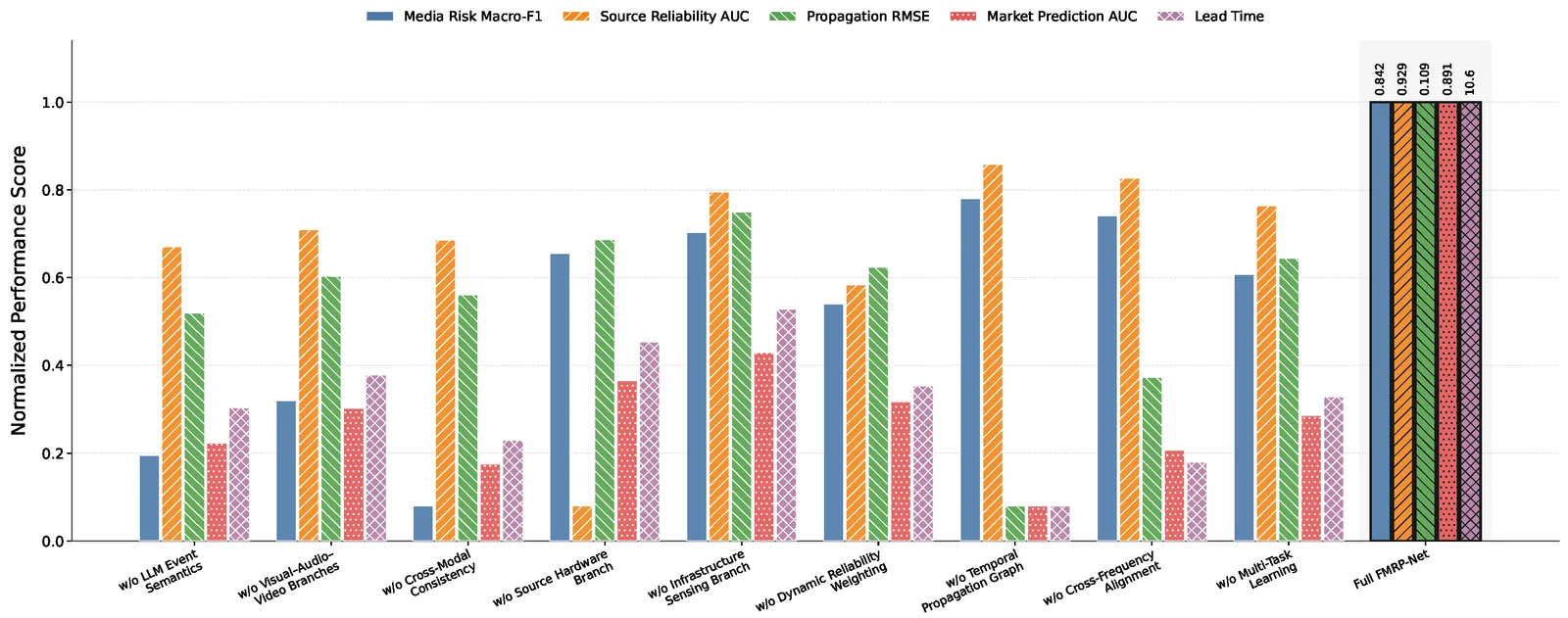
Normalized performance comparison of different ablation variants in media risk identification, source reliability assessment, propagation-scale prediction, market prediction, and early warning, with the complete FMRP-Net achieving the best overall performance.

As shown in Table 8 and Figure 6, the complete model achieved the best results for media risk identification, source reliability assessment, propagation-scale prediction, market prediction, and early warning, with values of 0.842, 0.929, 0.109, 0.891, and 10.6 min, respectively. After the pretrained financial-language event semantic component was removed, the media risk Macro-F1 decreased to 0.758, while the market prediction AUC decreased to 0.842. This result indicates that risk representations are weakened when event topics, sentiment intensity, and asset association semantics are unavailable. When the visual, audio, and video branches were removed, the media risk Macro-F1 decreased to 0.771, demonstrating that text alone cannot adequately identify image–text conflicts, audio–visual mismatches, or reused historical materials. After the cross-modal consistency mechanism was removed, the media risk Macro-F1 decreased further to 0.746, and the market prediction AUC reached only 0.839. Conflict identification across modalities was, therefore, shown to be essential for filtering misleading information. After the source hardware branch was removed, the source reliability AUC decreased substantially to 0.812, confirming that camera pattern noise, microphone fingerprints, and terminal motion characteristics provide important evidence for content provenance analysis. When the infrastructure sensing branch was removed, the market prediction AUC decreased to 0.855, and the warning lead time was reduced to 8.7 min. Platform traffic, power consumption, and network operating states were, therefore, shown to provide complementary indicators of market pressure.

From a theoretical perspective, different sources of uncertainty are constrained by different components of FMRP-Net. When dynamic reliability weighting was removed, the source reliability AUC decreased to 0.876, and the propagation prediction error increased to 0.127. Fixed weighting was, therefore, shown to be insufficient for suppressing missing, drifting, or abnormal sensing channels. The largest degradation in propagation-related performance was observed after removal of the temporal propagation graph. The propagation-scale prediction error increased to 0.153, the market prediction AUC decreased to 0.833, and the warning lead time was shortened to 6.9 min. This degradation occurred because conventional feature aggregation cannot preserve the topological dependencies and propagation order among users, content, devices, and platforms. When cross-frequency alignment was removed, the propagation prediction error increased to 0.139. Temporal misalignment among discrete media events, minute-level propagation records, second-level hardware signals, and high-frequency market sequences, therefore, weakens their effective associations. Performance degradation was also observed across all metrics after multi-task learning was removed, demonstrating that content risk, source reliability, propagation trends, and market prediction share informative representations and provide mutual constraints. In the complete FMRP-Net, event separability is enhanced through semantic encoding, misleading content is downweighted through cross-modal consistency, physical evidence is incorporated through hardware perception, and propagation dependencies are modeled using dynamic graphs. Asynchronous signals are subsequently coordinated through cross-frequency fusion. Propagation prediction errors are, therefore, reduced, market discrimination is improved, and the available early-warning time is extended.

### 4.6. Discussion

The results demonstrate that the practical value of FMRP-Net is not limited to improving market direction and volatility prediction metrics. Instead, media content, information propagation processes, content-source evidence, platform operating states, and market trading behavior are connected within an interpretable risk identification chain. In practical scenarios involving unexpected earnings announcements, regulatory penalties, major litigation, or executive changes at listed companies, relevant information is often first disseminated through announcements, news reports, short videos, or social media posts and may subsequently undergo concentrated reposting within a short period. Conventional approaches generally classify such information as positive or negative according to textual sentiment. However, false warnings may be produced by reused historical images, exaggerated headlines, audio–visual inconsistencies, and unverified reposted statements. In FMRP-Net, company identities, asset associations, cross-modal consistency, and device-source evidence are jointly considered to determine whether the content is genuinely related to the target stock. Propagation velocity, critical dissemination nodes, and cross-platform migration are then analyzed, allowing securities firms, fund risk-management departments, and market surveillance institutions to distinguish ordinary public-opinion fluctuations from high-risk events that may trigger abnormal price movements. The proposed framework can also be applied to minute-level risk monitoring during major policy announcements, industrial accidents, product recalls, and market-rumor propagation. For example, when a video of a corporate accident is rapidly disseminated across multiple platforms, the risk level of the associated stock or industry index can be increased in advance if the video source is assessed as credible, the propagation scale continues to grow, and synchronous increases are observed in market-data server requests, network traffic, order-book imbalance, and trading activity. Such warnings may support adjustments to trading limits, portfolio exposure reduction, market-making quotation protection, and abnormal trading reviews. Conversely, when highly emotional content originates from a suspicious source, reused materials are detected, and no synchronized changes are observed in market activity or infrastructure pressure, the corresponding warning weight can be reduced, thereby limiting false alarms caused by misleading information. The advantages achieved by the complete model in direction prediction, volatility estimation, and warning lead time demonstrate that market risk is not determined solely by historical prices. Instead, it is jointly influenced by information authenticity, propagation intensity, asset relevance, and trading-system conditions. FMRP-Net is, therefore, particularly suitable for event-driven real-time monitoring and can provide integrated support for institutions from information verification and risk localization to market response assessment. The experiment is event-local but does not omit the principal contemporaneous economic controls. The market branch includes the broad-market and industry-index returns recorded in Table 1, together with turnover, bid–ask spread, order-book depth, and imbalance; these variables condition predictions on market trend and liquidity before the event. The contribution being tested is the incremental sensing chain after those controls, rather than a claim that local media alone determines prices. Comprehensive global macroeconomic releases, foreign-market linkages, currency and commodity shocks, and latent institutional flows are outside the present identification window. The graph and gated-fusion formulation can accept such variables as context nodes without altering the proposed provenance–propagation mechanism; evaluating that extension with financial economists is a priority for cross-market work.

### 4.7. Limitation and Future Work

Several limitations remain in the present study. First, the collected data primarily cover specific securities markets, media platforms, and infrastructure environments. Substantial differences exist among countries in terms of trading mechanisms, information disclosure regulations, investor structures, and platform propagation patterns. Consequently, the applicability of the proposed model to cross-market and multilingual scenarios requires further validation. Second, hardware signals such as camera pattern noise, microphone fingerprints, server loads, and network states depend on relatively complete acquisition conditions. In real-world institutions, the availability and quality of these signals may be restricted by device heterogeneity, interface permissions, channel missingness, and privacy requirements. Third, the relationship between events and market movements may be jointly affected by latent factors such as macroeconomic policies, industry linkages, and institutional trading behavior. Although propagation processes and market responses can be jointly characterized by the current framework, not all statistical associations can be interpreted directly as causal effects. In addition, the interpretation of metaphorical expressions, domain-specific rumors, and rapidly evolving events by pretrained financial language models may remain imperfect. Future studies will extend the data sources to additional markets, languages, and event categories, followed by cross-regional, cross-platform, and cross-year validation. Privacy-preserving collaborative training may also be introduced to enable multiple institutions to utilize infrastructure operating states without exchanging raw data. Adaptive calibration, modality completion, and lightweight edge inference will be investigated to address sensing-channel missingness and device variation. Furthermore, causal inference, market mechanism constraints, and expert knowledge will be incorporated to improve the credibility of interpretations concerning risk propagation paths and price responses. Prospective evaluations in real-world trading surveillance environments will also be conducted to assess the practical value of the generated warnings for risk control, trading-limit adjustment, and abnormal-event management.

## 5. Conclusions

As information dissemination has expanded from text-based communication to multimedia forms, including news articles, images, audio recordings, short videos, and livestreams, market risk is no longer determined solely by historical price movements. Instead, it is jointly influenced by content authenticity, propagation velocity, asset relevance, and the operating conditions of trading infrastructures. FMRP-Net is proposed as a unified framework that integrates artificial intelligence-based semantic perception, hardware sensing evidence, dynamic propagation relationships, and market sequences. A complete analytical chain is thereby established, covering media risk identification, source reliability verification, propagation trend modeling, and short-term market response prediction. The principal innovations include the use of a pretrained financial language model and cross-modal consistency analysis to identify risk semantics and misleading content, the integration of content-acquisition device fingerprints and platform infrastructure states through dual-layer hardware reliability perception, and the characterization of dynamic relationships between media propagation and market variation through heterogeneous temporal graphs, cross-frequency alignment, and bidirectional coupling. Experimental results demonstrate that FMRP-Net achieved an Accuracy of 0.832, a Macro-F1 of 0.824, a ROC-AUC of 0.891, an MCC of 0.665, and a PR-AUC of 0.883 for market direction prediction over future horizons of 5, 15, 30, and 60 min, indicating a well-balanced performance in terms of Precision and Recall. In the volatility prediction task, an MAE of 0.0178, an RMSE of 0.0271, a MAPE of 12.46%, and an $R^{2}$ of 0.812 were obtained. The ablation results further showed that the complete model achieved a media risk Macro-F1 of 0.842 and a source reliability AUC of 0.929, while the propagation-scale prediction error was reduced to 0.109 and the average early-warning lead time reached 10.6 min. These findings confirm that artificial intelligence-driven multimodal sensing, hardware evidence integration, and propagation modeling can effectively improve the accuracy, stability, and practical applicability of market prediction and risk early warning.

## Figures and Tables

**Table 1:** Acquisition sensors and actual retained data volumes after quality control for different data types in the FMRP-Net dataset.

Data Type	Sensor Type	Data Volume
News, announcements, and social media text	Information acquisition sensor	1,286,420 records
News images and social media illustrations	Full-frame image sensor (Sony A7 IV, Sony Corporation, Tokyo, Japan)	184,760 images
Short videos and livestreaming clips	CMOS video sensor (DJI Osmo Action 4, DJI, Shenzhen, China)	32,480 clips
Speech and environmental audio	Wireless audio sensor (DJI Mic 2, DJI, Shenzhen, China)	28,650 clips
Terminal acceleration and angular velocity	Six-axis inertial sensor (Bosch BMI160, Bosch Sensortec GmbH, Reutlingen, Germany)	16,102,487 records
Content propagation nodes	Network propagation behavior sensor	2,764,820 nodes
Content propagation edges	Cross-platform relationship sensing sensor	8,936,470 edges
Server temperature and resource utilization	Server state sensor (HPE iLO/IPMI, Hewlett Packard Enterprise, Spring, TX, USA)	30,847,216 records
Server power consumption, current, and voltage	Intelligent power sensor (APC AP8959, APC by Schneider Electric, West Kingston, RI, USA)	15,283,604 records
Network traffic, latency, jitter, and packet loss	Network state sensor (Intel X710, Intel Corporation, Santa Clara, CA, USA)	30,421,778 records
Second-level market sequences	Quotation sensing sensor	41,974,286 records
Media event-level samples	Multi-source event synchronization sensor	96,315 samples

**Table 2:** Performance comparison of different methods on the short-term market direction prediction task. The results are averaged over the 5-, 15-, 30-, and 60-min forecasting horizons and are reported as the mean ± 95% confidence interval over repeated time-series experiments. The best results are shown in bold, and the second-best results are underlined. ↑ indicates that higher values are better.

Method	Accuracy ↑	Macro-F1 ↑	ROC-AUC ↑	MCC ↑	PR-AUC ↑
XGBoost	0.704±0.009	0.691±0.011	0.758±0.008	0.409±0.013	0.742±0.010
LSTM	0.731±0.008	0.719±0.010	0.781±0.009	0.463±0.012	0.767±0.009
Informer	0.752±0.007	0.740±0.009	0.804±0.008	0.505±0.011	0.791±0.008
FinBERT	0.742±0.009	0.733±0.010	0.798±0.009	0.486±0.013	0.785±0.010
Multimedia Transformer	0.768±0.007	0.756±0.008	0.823±0.007	0.537±0.010	0.812±0.008
DeepHawkes	0.746±0.008	0.732±0.010	0.807±0.008	0.493±0.012	0.794±0.009
Temporal Graph Network	0.781±0.007	0.770±0.008	0.838±0.007	0.563±0.010	0.826±0.008
Text–Market Fusion Transformer	0.794±0.006	0.783±0.007	0.851±0.006	0.589±0.009	0.839±0.007
Sensor–Market Fusion Transformer	0.803±0.006	0.792±0.007	0.862±0.006	0.607±0.009	0.851±0.007
PatchTST	0.789±0.006	0.780±0.007	0.847±0.006	0.579±0.009	0.837±0.007
TimesNet	0.798±0.006	0.789±0.007	0.856±0.006	0.596±0.009	0.846±0.007
Modality-aware Transformer	$\underline{0.812\pm 0.006}$	$\underline{0.804\pm 0.006}$	$\underline{0.872\pm 0.006}$	$\underline{0.624\pm 0.009}$	$\underline{0.864\pm 0.006}$
FMRP-Net	0.832±0.005	0.824±0.006	0.891±0.005	0.665±0.008	0.883±0.006

**Table 3:** Performance comparison of different methods on the future market volatility prediction task. The results are averaged over the 5-, 15-, 30-, and 60-min forecasting horizons and are reported as the mean ± 95% confidence interval over repeated time-series experiments. The best results are shown in bold, and the second-best results are underlined. ↑ indicates that higher values are better, whereas ↓ indicates that lower values are better.

Method	MAE ↓	RMSE ↓	MAPE (%) ↓	$R^{2}$ ↑	Spearman ↑
XGBoost	0.0268±0.0007	0.0389±0.0010	18.73±0.46	0.612±0.014	0.701±0.013
LSTM	0.0243±0.0006	0.0355±0.0009	17.14±0.42	0.657±0.013	0.729±0.012
Informer	0.0229±0.0006	0.0338±0.0008	16.02±0.39	0.691±0.012	0.754±0.011
FinBERT	0.0251±0.0007	0.0364±0.0009	17.62±0.44	0.641±0.014	0.718±0.013
Multimedia Transformer	0.0218±0.0005	0.0322±0.0008	15.38±0.36	0.721±0.011	0.778±0.010
DeepHawkes	0.0235±0.0006	0.0344±0.0008	16.47±0.40	0.676±0.012	0.744±0.011
Temporal Graph Network	0.0211±0.0005	0.0315±0.0007	14.92±0.35	0.739±0.010	0.791±0.010
Text–Market Fusion Transformer	0.0202±0.0005	0.0303±0.0007	14.28±0.33	0.757±0.010	0.806±0.009
Sensor–Market Fusion Transformer	0.0196±0.0004	0.0295±0.0007	13.87±0.31	0.771±0.009	0.817±0.009
PatchTST	0.0207±0.0005	0.0309±0.0007	14.63±0.34	0.748±0.010	0.799±0.009
TimesNet	0.0201±0.0005	0.0302±0.0007	14.19±0.33	0.759±0.010	0.808±0.009
Modality-aware Transformer	$\underline{0.0189\pm 0.0004}$	$\underline{0.0286\pm 0.0006}$	$\underline{13.35\pm 0.30}$	$\underline{0.786\pm 0.009}$	$\underline{0.829\pm 0.008}$
FMRP-Net	0.0178±0.0004	0.0271±0.0006	12.46±0.28	0.812±0.008	0.849±0.008

**Table 4:** Horizon-resolved FMRP-Net performance. Values are mean ± 95% confidence interval over event-cluster bootstrap resamples.

Horizon	Accuracy	Macro-F1	ROC-AUC	MAE	RMSE	MAPE (%)	$R^{2}$
5 min	0.856±0.006	0.849±0.006	0.910±0.005	0.0148±0.0003	0.0229±0.0005	10.08±0.24	0.856±0.007
15 min	0.840±0.006	0.833±0.006	0.900±0.005	0.0167±0.0004	0.0254±0.0006	11.62±0.26	0.831±0.008
30 min	0.824±0.007	0.817±0.007	0.886±0.006	0.0186±0.0004	0.0282±0.0006	13.07±0.29	0.798±0.009
60 min	0.808±0.008	0.797±0.008	0.868±0.007	0.0211±0.0005	0.0319±0.0007	15.07±0.34	0.763±0.010

**Table 5:** Source-reliability results under device-disjoint evaluation. ↑ indicates that higher values are better, whereas ↓ indicates that lower values are better.

Protocol	Reliability AUC ↑	Manipulation Macro-F1 ↑	EER (%) ↓
Standard event split	0.929±0.005	0.874±0.007	7.8±0.4
Device/model-disjoint split	0.897±0.007	0.842±0.009	10.6±0.6
Fingerprint-only, device-disjoint	0.811±0.011	0.752±0.012	17.4±0.9

**Table 6:** One-factor-at-a-time hyperparameter sensitivity on the chronological validation set. ↑ indicates that higher values are better, whereas ↓ indicates that lower values are better.

Factor	Value	Macro-F1 ↑	ROC-AUC ↑	RMSE ↓
Hidden width	128	0.813	0.882	0.0281
	256	**0.821**	**0.889**	**0.0275**
	384	0.819	0.887	0.0277
Dropout	0.1	0.817	0.885	0.0278
	0.2	**0.821**	**0.889**	**0.0275**
	0.3	0.815	0.883	0.0280
Kernel half-life	5 min	0.816	0.884	0.0279
	15 min	**0.821**	**0.889**	**0.0275**
	30 min	0.814	0.882	0.0281

**Table 7:** Computational cost under the common hardware protocol. Inference is per event at batch size 1.

Model	Parameters (M)	Training (h)	Inference (ms)	Peak GPU (GB)
Text–Market Fusion Transformer	132.4	5.6	14.8	12.6
Sensor–Market Fusion Transformer	146.1	7.2	18.9	15.8
FMRP-Net	286.7	12.4	43.6	34.7

**Table 8:** Ablation results for the key components of FMRP-Net. The results are reported as the mean ± 95% confidence interval over repeated time-series experiments. ${\mathrm{RMSE}}_{\mathrm{prop}}$ denotes the prediction error for future propagation scale, while Lead Time denotes the average early-warning time for high-risk events. The best results are shown in bold. ↑ indicates that higher values are better, whereas ↓ indicates that lower values are better.

Model Variant	Media Risk Macro-F1 ↑	Source Reliability AUC ↑	${\mathrm{RMSE}}_{\mathrm{prop}}$ ↓	Market Prediction AUC ↑	Lead Time (min) ↑
w/o Pretrained Financial-Language Semantics	0.758±0.010	0.887±0.008	0.132±0.004	0.842±0.007	7.8±0.4
w/o Visual–Audio–Video Branches	0.771±0.009	0.892±0.008	0.128±0.004	0.847±0.007	8.1±0.4
w/o Cross-Modal Consistency	0.746±0.011	0.889±0.008	0.130±0.004	0.839±0.008	7.5±0.5
w/o Source Hardware Branch	0.806±0.008	0.812±0.012	0.124±0.004	0.851±0.007	8.4±0.4
w/o Infrastructure Sensing Branch	0.811±0.008	0.903±0.007	0.121±0.003	0.855±0.007	8.7±0.4
w/o Dynamic Reliability Weighting	0.794±0.009	0.876±0.009	0.127±0.004	0.848±0.007	8.0±0.4
w/o Temporal Propagation Graph	0.819±0.007	0.911±0.007	0.153±0.005	0.833±0.008	6.9±0.5
w/o Cross-Frequency Alignment	0.815±0.008	0.907±0.007	0.139±0.004	0.841±0.008	7.3±0.5
w/o Multi-Task Learning	0.801±0.008	0.899±0.008	0.126±0.004	0.846±0.007	7.9±0.4
Full FMRP-Net	0.842±0.006	0.929±0.005	0.109±0.003	0.891±0.005	10.6±0.3

## Data Availability

The reproducibility package and related data are available from the corresponding author upon reasonable request.
